# Peripheral nerve field stimulation for chronic low back pain: a systematic review

**DOI:** 10.1097/PR9.0000000000001488

**Published:** 2026-10-05

**Authors:** Reza Hazrati, Elie Ruest, Baraa Dabboucy, Sobee Dansereau, Chloe Salamon, Shirley Fecteau, Michel Prudhomme

**Affiliations:** a Neurosurgery Service, Department of Surgery, CHU de Québec, Université Laval, Québec, QC, Canada; b Department of Psychiatry and Neuroscience, Faculty of Medicine, Université Laval, Québec, QC, Canada

**Keywords:** Peripheral nerve field stimulation, Chronic low back pain, Subcutaneous stimulation, Neuromodulation, Systematic review

## Abstract

Chronic low back pain (CLBP) presents a major challenge in pain management, often persisting despite standard therapies. Peripheral nerve field stimulation (PNFS), also known as subcutaneous stimulation, offers a minimally invasive neuromodulation approach that may improve outcomes for these patients. This systematic review evaluates the evidence on PNFS, alone or combined with spinal cord stimulation (SCS), for CLBP, focusing on pain relief, functional outcomes, and safety. Following Preferred Reporting Items for Systematic Reviews and Meta-Analyses guidelines, databases including Cochrane, Embase, MEDLINE (PubMed), and the Université Laval Library were searched for studies from January 2014 to April 2025. Nineteen studies (randomized controlled trials, cohort studies, and case series) were included. Outcomes assessed included pain intensity, disability (Oswestry Disability Index), quality of life (36-Item Short Form Health Survey, EuroQol 5-Dimension), and adverse events. Across study-level estimates, PNFS monotherapy most commonly produced approximately 40% to 60% reductions in pain intensity, although reported effects varied across studies and were not pooled because of heterogeneity. Functional gains included reduced disability and improved mobility. Quality of life measures and medication use often improved. Peripheral nerve field stimulation combined with SCS yielded mixed results, providing benefits particularly for patients unresponsive to SCS alone. Reported complication rates were generally modest, with infections and lead migrations being the most common. Peripheral nerve field stimulation appears to be a promising and generally well-tolerated option for selected patients with CLBP, although evidence certainty remains limited, and larger controlled studies are needed.

## Abstract 2

Supplemental Digital Content is Available in the Text.

This systematic review synthesizes 19 studies on peripheral nerve field stimulation for chronic low back pain, highlighting pain relief, functional improvements, safety, and research needs.

## 1. Introduction

Chronic low back pain (CLBP) is a significant global health burden, affecting between 15% and 45% of adults annually and ranking among the leading causes of disability and work absenteeism worldwide.^16^ It can profoundly affect multiple domains of daily life, including work productivity, physical activity, sleep, and overall quality of life (QOL).^1^ Chronic low back pain includes a heterogeneous group of conditions, such as persistent spinal pain syndrome (PSPS), degenerative spinal disease, occupational or lifestyle-related back pain, and sacroiliac joint (SIJ) dysfunction.^16,24^ Sacroiliac joint–related pain is a common and often difficult-to-treat contributor to low back pain and has shown responsiveness to neuromodulation in selected studies.^3,33^ Despite the availability of conservative, pharmacological, interventional, and surgical options, CLBP remains challenging for both patients and clinicians because symptoms may persist despite conventional treatment.^27^

Spinal cord stimulation (SCS) is an established neuromodulation technique that has been used since the late 1960s for the management of chronic neuropathic pain, including PSPS and CLBP.^6,23^ Conventional low-frequency, or tonic, SCS typically produces paresthesia intentionally targeted to overlap the painful region, using this sensory coverage to guide stimulation programming and parameter selection. Although the gate-control theory proposed by Melzack and Wall remains an important historical and mechanistic framework for paresthesia-based stimulation, the analgesic effects of SCS are now understood to involve broader modulation of nociceptive processing through segmental spinal circuits, dorsal column and dorsal horn activity, supraspinal networks, and neurochemical mechanisms, including descending modulatory pathways.^19,27,32,34^ Newer paradigms such as high-frequency and burst stimulation further support these broader mechanisms, as clinically meaningful analgesia may occur even in the absence of perceived paresthesia.^27,32,34^ Despite advances in SCS technology, some patients continue to experience incomplete relief of axial low back pain, which has motivated interest in complementary or alternative neuromodulation strategies such as peripheral nerve field stimulation (PNFS).^27,34^

Peripheral nerve field stimulation, also referred to as subcutaneous electrical stimulation, has emerged as a complementary or stand-alone neuromodulation technique for chronic low back and related regional pain syndromes.^36^ In contrast to peripheral nerve stimulation, PNFS does not require direct targeting of a named peripheral nerve. Instead, 1 or more leads are placed in the subcutaneous tissue over the area of maximal pain and connected to an implantable pulse generator positioned away from the painful region.^13^ This approach may simplify implantation and reduce the risk of direct peripheral nerve injury. PNFS is also minimally invasive, reversible, and adaptable, and it can be used alone or in combination with SCS in selected patients with persistent axial pain.^12,40,43^ Its potential utility has also been described in conditions such as SIJ-related pain, which may coexist with or contribute to CLBP.^3,26,33^

Clinical studies have reported reductions in pain intensity and improvements in function and QOL after PNFS in patients with CLBP, PSPS, and sacroiliac pain. For example, Eldabe et al.^7^ reported that patients receiving PNFS plus optimized medical management achieved greater pain relief at 9 months than those receiving optimized medical management alone. However, the available literature remains heterogeneous in study design, patient selection, outcome measures, follow-up duration, and reporting of stimulation parameters. Although newer dedicated PNS platforms and restorative neurostimulation approaches have expanded the neuromodulation landscape for CLBP, classical PNFS/subcutaneous stimulation remains a distinct technique with an evidence base that warrants focused synthesis. Unlike broader reviews of peripheral nerve stimulation across multiple pain conditions, anatomical targets, and stimulation approaches, such as the review by Deer et al.,^5^ this review focuses specifically on PNFS/subcutaneous stimulation for CLBP. The objective of this systematic review is to summarize recent evidence on PNFS for CLBP, including pain reduction, disability, QOL, trial protocols, programming parameters, complications, and its role as monotherapy or as an adjunct to SCS, while identifying remaining clinical and methodological knowledge gaps.

## 2. Materials and methods

### 2.1. Search strategy

A systematic literature search was conducted in accordance with the Preferred Reporting Items for Systematic Reviews and Meta-Analyses guidelines^25^ to identify studies published in English or French between January 2014 and April 2025. Searches were performed between April 5 and April 8, 2025, in Cochrane, Embase, MEDLINE (PubMed), and the Université Laval Library. Search strategies were adapted for each database using combinations of free-text terms related to the intervention and condition, including “peripheral nerve field stimulation,” “subcutaneous stimulation,” “subcutaneous electrical stimulation,” “peripheral nerve stimulation,” “low back pain,” “chronic low back pain,” “lumbar pain,” “chronic lumbar pain,” “failed back surgery syndrome,” “FBSS,” “persistent spinal pain syndrome,” “PSPS,” and “sacroiliac joint pain.” Boolean operators were used to combine intervention-related terms with low back pain–related terms, and results were limited to English- or French-language publications from 2014 onward. The database-specific search strategies are provided in Supplementary Table 1 (available at https://links.lww.com/PR9/A440). Additional relevant articles were identified by manually searching the reference lists of retrieved papers.

### 2.2. Study selection

After duplicates were removed, 3 independent reviewers screened all titles and abstracts identified through the search. Studies that met the predefined inclusion criteria were retrieved for full-text assessment. A second screening of full-text articles was then conducted using the same eligibility criteria to determine final inclusion. In cases of disagreement, a fourth independent reviewer was consulted to resolve discrepancies. Studies were included if they were published in 2014 or later (up to April 2025), written in English or French, and reported quantitative data on the effectiveness of subcutaneous stimulation (PNFS alone or in combination with SCS) on pain intensity and/or related functional outcomes or QOL in CLBP. Programming parameters of stimulation were also reviewed. Review articles and articles measuring the effectiveness of subcutaneous stimulation on regions other than the lower back were excluded.

### 2.3. Data extraction

Data from each included study was extracted by 3 authors independently after full-text review, and any discrepancies were resolved by consensus. Details were collected on study design, patient demographics, pain etiology, stimulation parameters (eg, frequency, pulse width, amplitude), lead placement and orientation, outcome measures (pain reduction, functional improvement, QOL scores), inclusion/exclusion criteria, and any reported complications or adverse events. The primary outcomes of interest were pain intensity reduction, improvement in QOL, and functional score improvement compared with baseline.

### 2.4. Risk of bias and quality appraisal

The risk of bias and quality of included studies were assessed using validated tools. The Cochrane risk-of-bias tool for randomized trials (RoB2) was used for all included randomized controlled trials (RCTs).^35^ A single RoB2 assessment was used to evaluate each randomized trial together with its related subanalyses and long-term follow-up studies (Fig. 1A). Domain-level RoB2 judgments and brief rationales for each randomized trial are provided in Supplementary Table 2 (available at https://links.lww.com/PR9/A440). For nonrandomised cohort studies, the Newcastle–Ottawa Scale was used to appraise study quality in selection, comparability, and outcome assessment (Fig. 1B).^44^ The Joanna Briggs Institute Critical Appraisal checklists were used to critically assess the quality and reliability of included case reports and case series. Included case series and case reports all met most of the core quality criteria set by the Joanna Briggs Institute tools (Fig. 1C and D).^21,22^ Overall, the quality of evidence was limited by the prevalence of observational designs and small sample sizes. The included RCTs generally had high risk of bias, most were unblinded because of the nature of the intervention, whereas the observational studies scored moderate on the Newcastle–Ottawa Scale. This indicates a moderate to high risk of bias in the body of evidence, and the findings should be interpreted with appropriate caution.

**Figure 1.**
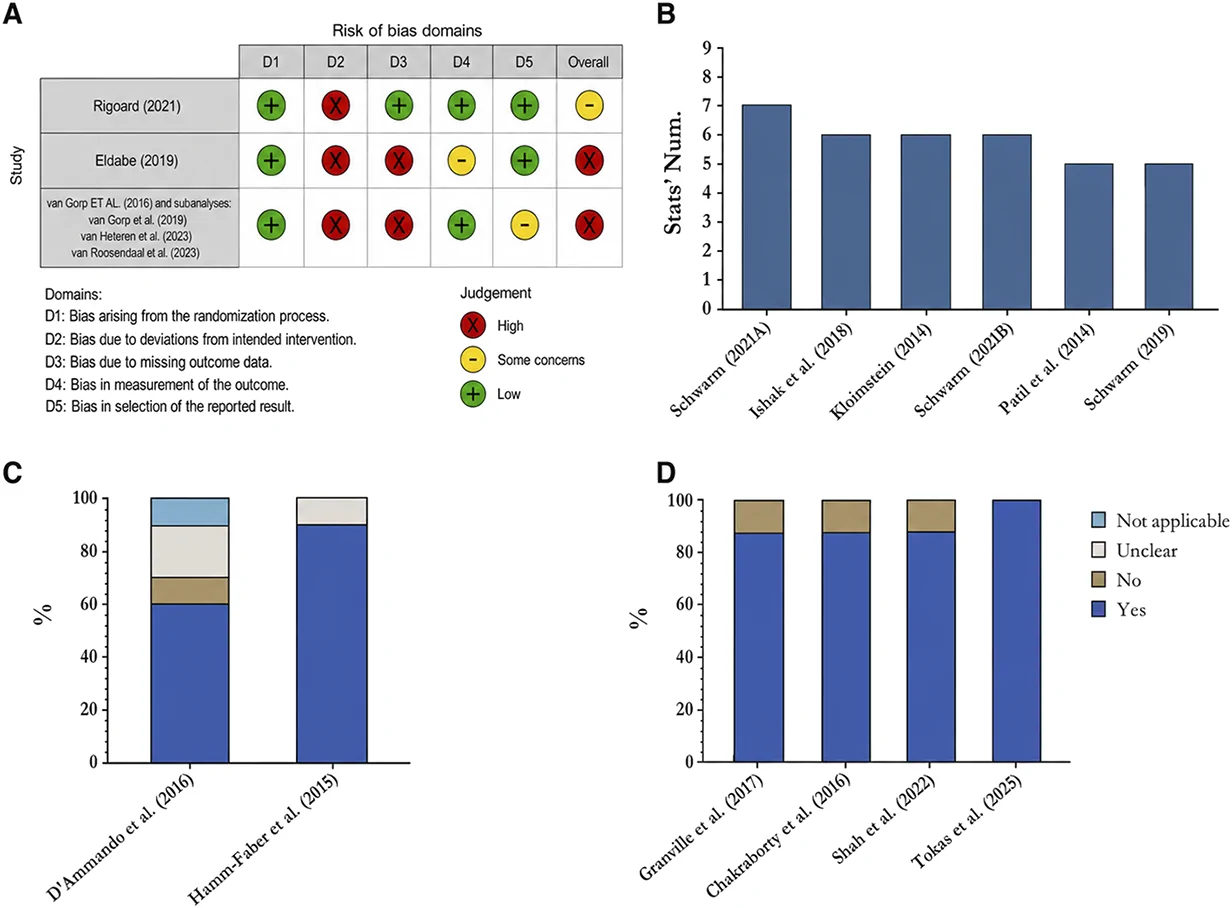
Quality assessment of included studies. Panel A is a traffic-light plot illustrating the risk-of-bias judgments for each randomized controlled trial across the 5 domains of the Cochrane RoB 2 tool (D1: randomization, D2: deviations from intended interventions, D3: missing outcome data, D4: outcome measurement, and D5: selection of the reported result).^17^ Panel B summarizes the methodological quality of cohort studies using the NOS; bars indicate the number of stars earned in the selection, comparability, and outcome domains (maximum 9 stars). Panel C depicts the JBI Critical Appraisal Checklist ratings for case series, showing the proportion of checklist items scored “Yes,” “No,” “Unclear,” or “Not applicable.” Panel D presents the same JBI ratings for case reports. Higher proportions of green or “Yes” responses reflect stronger methodological quality, whereas red or low scores highlight potential bias. JBI, Joanna Briggs Institute; NOS, Newcastle–Ottawa Scale; RoB, risk-of-bias.

### 2.5. Missing data

None of the included studies reported significant issues with missing outcome data that would impair our analysis. All studies provided sufficient data on primary outcomes for extraction. However, some secondary outcome parameters (eg, QOL, medication reduction, or functional capacity) were not uniformly reported across all studies. Therefore, we included and analyzed these outcomes only when explicitly reported by the original studies. Where data were unclear or lacking specific statistical details (eg, standard deviations or confidence intervals), we noted those limitations accordingly. In general, no imputation of missing data was required.

### 2.6. Synthesis strategy

Because of substantial clinical and methodological heterogeneity across study designs, patient populations, stimulation approaches, comparators, outcome definitions, follow-up durations, and reporting formats, a meta-analysis was not performed. Potentially comparable subsets were reviewed with statistical support, including PNFS plus optimized medical management vs optimized medical management alone and PNFS added to SCS vs SCS alone. However, these contrasts were either represented by a single randomized trial dataset or differed substantially in study design, treatment context, follow-up timing, and outcome reporting, limiting the validity of pooled estimates. Therefore, quantitative heterogeneity statistics such as I^2^ and τ^2^ were not calculated, as they would not be meaningful for single-study contrasts or clinically noncomparable pooled models. Results were synthesized narratively and tabulated by outcome domain. Effect measures were reported as presented in the original studies, including absolute pain scores, percentage change from baseline, responder rates, Oswestry Disability Index (ODI) and QOL score changes, and complication rates. The certainty of evidence for critical outcomes, including pain intensity, disability assessed with the ODI, and health-related QOL assessed with EuroQol 5-Dimension (EQ-5D), was evaluated using the Grading of Recommendations Assessment, Development, and Evaluation framework and summarized in a Summary of Findings table.

The review protocol was not prospectively registered, and no formal protocol was published before completion of the review.

## 3. Results

### 3.1. Article selection

The initial database search (Cochrane, Embase, Medline [PubMed], and the library of Université Laval) yielded 290 publications. After removal of duplicates, 196 unique articles remained. Based on titles and abstracts, most were excluded for not meeting inclusion criteria. Most of the excluded articles were omitted because they did not assess PNFS in the low back region, were unavailable in full text, or were published in a language other than English or French. Ultimately, 19 studies published between 2014 and 2025 fulfilled all criteria and were included in this systematic review (Fig. 2).

**Figure 2.**
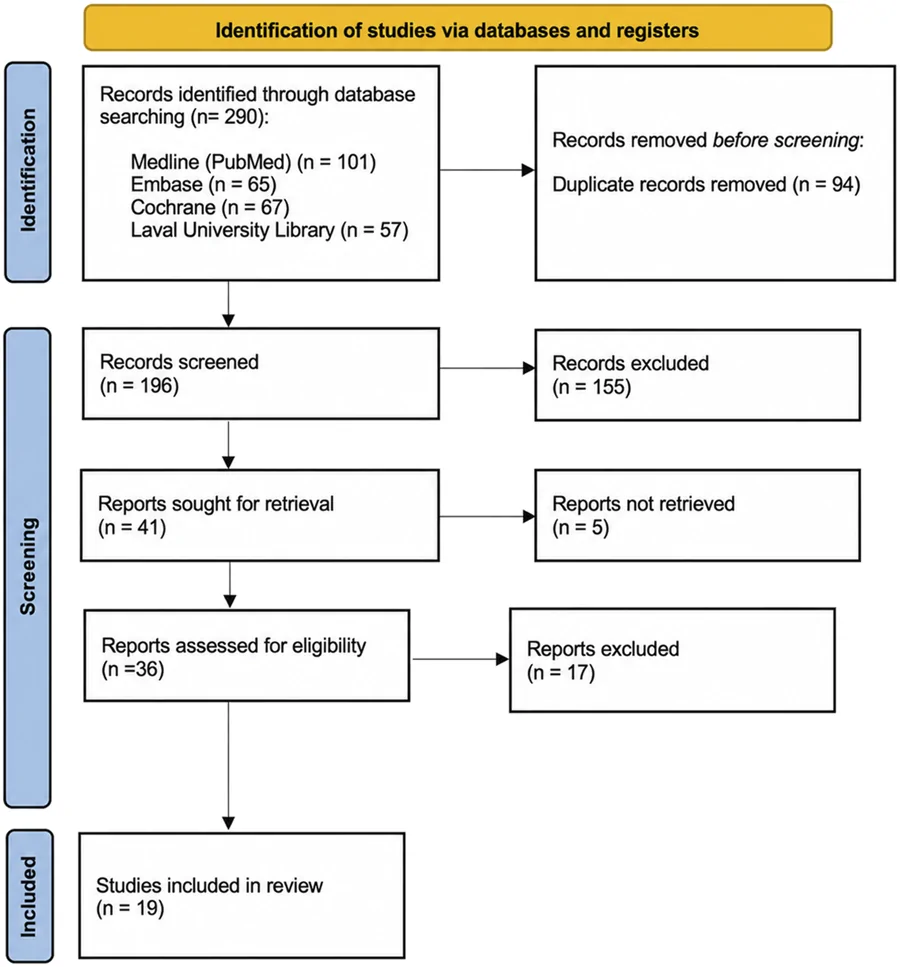
PRISMA flow diagram. PRISMA flowchart describing the study selection process. Boxes detail the number of records identified, screened, assessed for eligibility, and included in qualitative and quantitative analyses, with reasons for exclusion at each stage. PRISMA, Preferred Reporting Items for Systematic Reviews and Meta-Analyses.

### 3.2. Characteristics of included studies

A variety of study designs were represented among the 19 included studies: 3 were randomized controlled trials, with some reporting extended follow-up or subgroup analyses in separate publications for a total of 6 RCT-related publications, 7 were prospective or retrospective cohort studies, and 6 were case reports or small case series. Although 11 studies were about PNFS as a stand-alone therapy, 8 studied PNFS in addition to SCS (either as an add-on to existing SCS therapy or vs SCS alone in a comparative design). Sample sizes varied widely, from single-patient case reports to the largest multicenter cohort of 117 patients.^6^ All studies were published between 2014 and 2025, reflecting relatively recent data in the field. Table 1 provides a summary of the included studies, detailing their design, sample sizes, and interventions.

**Table 1:** Summary of included studies and patient demographics.

Study	Publication year	Study type	PNFS or PNFS + SCS	Total patients (n)	Males (n)	Females (n)	Implanted after trial phase
Eldabe et al. (2019)^7^	2019	RCT	PNFS	116	50	66	46
Rigoard et al. (2021)^28^	2021	RCT	SCS or PNFS + SCS	14	3*	10*	13
Van Gorp et al. (2019)^39^,†	2019	RCT (follow-up)	SCS or PNFS + SCS	100	NR	NR	65
Van Gorp et al. (2016)^40^,†	2016	RCT	SCS or PNFS + SCS	100	NR	NR	65
Van Heteren et al. (2023)^41^,†	2023	RCT (subanalysis)	SCS or PNFS + SCS	100	NR	NR	65
Van Roosendaal et al. (2023)^42^,†	2023	RCT (subanalysis)	SCS or PNFS + SCS	100	NR	NR	65
Ishak et al. (2018)^12^	2018	Prospective cohort	PNFS	26	8	18	13
Kloimstein et al. (2014)^14^	2014	Prospective cohort	PNFS or PNFS + SCS	117	NR	NR	105
Patil et al. (2014)^26^	2014	Retrospective cohort	PNFS	10	6	4	10
Schwarm et al. (2021)^30^,‡	2021	Prospective cohort	PNFS	39	21	18	30
Schwarm et al. (2021)^29^,‡	2021	Prospective cohort	PNFS	41	22	19	32
Schwarm et al. (2019)^31^,‡	2019	Retrospective cohort	PNFS	25	13	12	20
Van Gorp et al. (2020)^38^	2020	Retrospective cohort	PNFS	54	NR	NR	54
D'Ammando et al. (2016)^4^	2016	Case series	PNFS	22	11	11	22
Hamm-Faber et al. (2015)^11^	2015	Case series	PNFS or PNFS + SCS	11	5	6	11
Chakrabortty et al. (2016)^3^	2016	Case report	PNFS	2	1	1	2
Granville et al. (2017)^9^	2017	Case report	PNFS	1	0	1	1
Shah et al. (2022)^33^	2022	Case report	PNFS	1	0	1	1
Tokas et al. (2025)^37^	2025	Case report	PNFS	1	0	1	1

This table lists the author and publication year, study design, type of intervention (PNFS alone vs PNFS combined with SCS), total number of patients, numbers of males and females, and the number of patients proceeding to permanent implantation.

Several publications represent follow-up or secondary analyses of overlapping cohorts. Therefore, patient numbers in Table 1 should not be summed across rows. Aggregate population counts in the Results were calculated after accounting for overlapping cohorts where identifiable.

* Sex distribution reported for the implanted/analyzed cohort rather than the full randomized sample.

† Publications derived from the same multicenter randomized cohort; rows are retained because they report different timepoints, follow-up analyses, subgroup analyses, or stimulation-parameter analyses and should not be interpreted as independent cohorts.

‡ Publications from overlapping single-center Schwarm cohorts; rows are retained because they report different analyses or outcome domains and should not be summed as independent cohorts.

NR, not reported; PNFS, peripheral nerve field stimulation; RCT, randomized controlled trial; SCS, spinal cord stimulation.

### 3.3. Measured outcomes

Various scores were used to analyze a multitude of outcomes among included articles; however, only the most used ones were analyzed. Out of the 19 studies included in this review, 18 assessed pain reduction using a visual analogue scale (VAS) or numeric rating scale (NRS), 9 used the ODI to rate disability among patients, and 5 studies used the 36-Item Short Form Survey (SF-36) score to assess global QOL. Other analyzed outcomes include complication rates and stimulation parameters.

### 3.4. Population characteristics

Across all 19 studies, a total of 526 patients were evaluated, including all patients in control groups and subgroups, with overlapping cohorts counted only once. Four publications^39–42^ reported on the same multicenter RCT cohort at different timepoints or in subgroup analyses, and 3 publications^29–31^ reported on overlapping cohorts from a single center. These overlapping cohorts are flagged with superscript markers in the relevant tables to clarify that they represent follow-up or secondary analyses rather than independent patient cohorts. Of these 526 patients, 372 underwent implantation of a neuromodulation system (PNFS alone or combined SCS + PNFS) after a successful trial stimulation to treat lower back pain. Therefore, 71% of the patients had a successful trial and received a permanent implant. Specifically, 178 patients were implanted with a PNFS system, and 194 patients were included in studies comparing SCS or PNFS alone vs PNFS + SCS combination therapy. The sex distribution was not uniformly reported and was sometimes provided only for analyzed or implanted subgroups rather than the full study population. Therefore, sex distribution was summarized descriptively and marked as NR in Table 1 when not clearly reported for the corresponding cohort. Patients' age ranged from 30 to 86 years old. The most common underlying causes of chronic low back pain included PSPS, SIJ pain, herniated discs, and lumbar spinal canal stenosis or postoperative fibrosis. All included studies required patients to have chronic pain for at least 3 to 6 months, and in most cases for over 1 year, before considering neurostimulation.

### 3.5. Trial stimulation

Nearly all studies implemented a temporary trial stimulation period before permanent implant (Table 2). The duration of this trial phase varied widely between studies. In some cases, the trial was as short as 3 to 7 days,^29–31^ which was sufficient to evaluate efficacy. Other studies used longer trial periods (eg, 2–4 weeks),^12^ and the SubQStim study reported trial phases of up to 36 days because of specific national regulations in Belgium,^7^ requiring an extended evaluation period before permanent implantation. Patients who reported significant pain relief (typically ≥50% reduction) and/or functional improvement during the trial were offered permanent PNFS implantation.

**Table 2:** Stimulation parameters reported in included studies.

Study	Intervention	Leads/configuration	Trial duration	Stimulation settings (amplitude, frequency, PW)
Eldabe et al. (2019)^7^	PNFS + OMM vs OMM alone	Up to 2 percutaneous cylindrical leads (1 × 8 Standard or Pisces Quad Plus); typically 2 leads placed vertically or horizontally in painful area; mostly nonrechargeable IPG	Mean trial 16 ± 8.7 d (range 4–36; longer in Belgium because of regulation)	Mean frequencies were set between 51.3 Hz and 65.3 Hz. Program 1 was set with a mean PW of 335.3 μs ± 87.9 and mean amplitude of 2.3 V ± 2.3. Program 2 was set with a mean PW of 320.4 μs ± 86.4 and mean amplitude of 2.9 V ± 2.4
Rigoard et al. (2021)^28^	PNFS + SCS vs SCS alone	1 Octad lead or 2 Quad leads added to standard SCS Octad epidural lead	No separate PNFS trial: PNFS implanted at baseline in “SCS + PNFS”; control received PNFS at 4 mo	PNFS frequency 40–90 Hz; PW 210–450 μs; amplitude 0.1–10.5 V
van Gorp et al. (2019)^39^,*	PNFS + SCS vs SCS.	Bilateral Quad Plus leads placed over CLBP area; IPG: Prime Advanced 37702 or Restore Advanced 37713	SCS trial ≥1 wk before randomization; no separate PNFS trial during RCT.	PNFS: 33.9 Hz, 366.1 µs, 3.3 V (means). Epidural: 34.0 Hz, 362.6 µs, 3.3 V (means)
van Gorp et al. (2016)^40^,*	PNFS as add-on to SCS vs SCS alone in PSPS.	1 or 2 Quad Plus subcutaneous leads (vertical, horizontal, or diagonal in painful back area), combined with epidural Octad lead for SCS	SCS trial ≥1 wk; then randomization; PNFS ON vs OFF for 3 mo	PNFS frequency 30 Hz; PW and amplitude individually optimized; field, flow, or triangular stimulation used
van Heteren et al. (2023)^41^,*	SCS + PNFS vs SCS.	Subcutaneous leads connected to the same IPG as SCS and placed over the painful low back area	1-wk SCS trial; PNFS implanted at the definitive implant if back pain response to SCS <50%	Tonic stimulation protocol referenced. Not numerically reported in this paper
van Roosendaal et al. (2023)^42^,*	SCS + PNFS vs SCS (subanalysis of van Heteren et al. RCT)	PNFS: 1–2 subcutaneous quadripolar leads placed horizontally/vertically/diagonal	Same cohort as van Heteren et al.	PNFS default: 30 Hz, PW 180 µs, ≤5 V (↑ PW if inadequate, up to 390 µs)
Ishak et al. (2018)^12^	PNFS for refractory CLBP.	2 quadripolar subcutaneous leads (Octrode/Quad Plus) in low back region	2–3 wk	Frequency: 50–80 Hz; pulse width: 450 μs; amplitude: 2.5–5 V
Kloimstein et al. (2014)^14^	PNFS (with/without SCS)	Subcutaneous leads placed at site of maximum pain	≥7 d trial (105/118 successful, ≥50% relief)	Parameters monitored but not reported
Patil et al. (2014)^26^	PNFS for SIJ.	8-contact leads, placed unilaterally (n = 2) or bilaterally (n = 8) over most painful area	Trial 4–5 d; permanent if ≥ 50% relief	Permanent: amplitude 1–8 V; frequency 40 ± 10 Hz; PW median 360 μs
Schwarm et al. (2021)^30^,†	PNFS for CLBP	2 percutaneous quadripolar leads (same technique as Schwarm 2021)	3–7 d PNFS trial; permanent if ≥50% relief	PNFS trial: 60 Hz, 1000 μs, 0.5–8.0 V; permanent stimulation same settings with patient-adjusted amplitude
Schwarm et al. (2021)^29^,†	PNFS for CLBP, stratified by TENS response	2 percutaneous quadripolar leads (Pisces-Quad 3487A) connected to Restore Ultra IPG.	PNFS trial: 3–7 d (after TENS 3–5 d)	PNFS trial: 60 Hz, 1000 μs, 0.5–8.0 V; permanent stimulation same settings with patient-adjusted amplitude
Schwarm et al. (2019)^31^,†	PNFS for CLBP	2 parallel percutaneous quadripolar leads (≈15 × 15 cm pain field coverage)	PNFS trial 3–5 d; implantation if ≥ 50% pain relief (20/25 succeeded)	Trial: 60 Hz, 1000 μs, 0.5–6.0 V; permanent: same frequency and pulse width, amplitude adjusted individually
D'Ammando et al. (2016)^4^	PNFS for chronic neuropathic pain (various etiologies incl. PSPS, PHN, post-thoracotomy pain)	Number of electrodes adapted to painful area; Boston Scientific leads; rechargeable IPG	No prior trial phase; direct permanent implantation	PW: 210–310 μs; frequency: 20–30 Hz; mean current: 3.2 mA
Hamm-Faber et al. (2015)^11^	SCS alone, PNFS alone, or combined SCS + PNFS.	SCS: 8-contact Octad (epidural); PNFS: 4-contact Quad Plus lead(s), placed mid back pain area with cross-lead communication	Trial period before permanent implant, based on ≥50% pain relief	Multiple programming modes described (flow, field, triangular) but no exact amplitude/frequency/PW values reported
Chakrabortty et al. (2016)^3^	PNFS for SIJ pain	2 parallel permanent 8-contact subcutaneous leads over painful SIJ	1-wk PNFS trial before permanent implant	Amplitude/frequency/PW not reported
Granville et al. (2017)^9^	Bilateral PNFS for coccygeal pain	Initial trial: single 8-contact subfascial lead in sacral fascia; permanent: 2 parallel 8-contact leads placed bilaterally in posterior sacral fascia with cross-lead communication	10-d PNFS trial; then 30-d stimulation-free interval; followed by permanent implantation	Example permanent settings: left lead (+2, +3/−5, −6), right lead (+10, +11/−13, −14). Exact amplitude, frequency, and PW not reported
Shah et al. (2022)^33^	PNFS for bilateral SIJ pain	2 Octrode percutaneous leads placed subcutaneously, parallel to bilateral SIJ	Trial: 8 d (4 d SCS: ineffective, 4 d PNFS: effective)	Effective parameters not numerically reported
Tokas et al. (2025)^37^	PNFS for chronic refractory coccydynia	Cylindrical percutaneous electrodes placed bilaterally at SIJ area, IPG in right gluteal pocket	Trial with externalized PNFS leads (duration not specified)	Parameters not detailed

Overview of trial design and stimulation settings for studies reporting relevant stimulation-parameter data. The table summarizes the number and configuration of subcutaneous leads, the duration of the trial phase before permanent implantation, and typical stimulation parameters (amplitude, frequency, and pulse width), where it is mentioned.

Rows sharing the same superscript should not be interpreted as independent patient cohorts or summed across rows.

* Publications derived from the same multicenter randomized cohort; rows are retained because they report different timepoints, subgroup analyses, or stimulation-parameter data.

† Publications from overlapping single-center Schwarm cohorts; rows are retained because they report different analyses or outcome domains.

CLBP, chronic low back pain; IPG, implantable pulse generator; OMM, optimized medical management; PHN, postherpetic neuralgia; PNFS, peripheral nerve field stimulation; PRI, patient review instrument; PSPS, persistent spinal pain syndrome; PW, pulse width; RCT, randomised controlled trial; SCS, spinal cord stimulation; SIJ, sacroiliac joint; TENS, transcutaneous electrical nerve stimulation; VAS, visual analogue scale.

### 3.6. Programming parameters

The included studies reported a range of stimulation programming parameters for PNFS, typically individualized per patient. In general, stimulation frequencies ranged from 20 Hz up to 90 Hz across studies^4,28^ (Table 2). Many clinicians favored using relatively lower frequencies (30–60 Hz) for long-term PNFS, aiming to maintain analgesia without excessive battery drain. Pulse widths were in the range of 200 to 1000 microseconds, adjusted based on patient comfort and the stimulated nerve field. Amplitude (voltage) settings varied widely depending on patient perception and impedances, usually starting around 0.5 to 1 V and being titrated up to as high as 10 V in some cases until therapeutic paresthesia covered the pain region. These parameters were often fine-tuned during follow-up visits: clinicians would increase or decrease the amplitude and pulse width to optimize pain relief while minimizing any uncomfortable sensations. Some studies explicitly noted that programming was customized over time, for instance, if a patient habituated to the sensation, the team might gradually increase amplitude or change pulse width to recapture pain control. The ability to tailor frequency, pulse width, and amplitude for each patient is 1 advantage of PNFS, as it allows optimization of the therapy to balance efficacy and side effects. Despite variations, most patients were ultimately programmed within relatively similar parameter ranges, reflecting a convergence toward what is effective for subcutaneous field stimulation. Table 2 summarizes stimulation parameters when reported in the included studies.

### 3.7. Pain reduction outcomes

#### 3.7.1. Peripheral nerve field stimulation as stand-alone therapy

A reduction in CLBP intensity was reported in 18 included studies. Eleven of these studies focused on PNFS used as a stand-alone therapy (without concurrent SCS) and consistently found notable pain improvements from baseline (Table 3, Fig. 3). Among PNFS monotherapy studies reporting percentage pain reduction or sufficient data to estimate it, study-level reductions most commonly ranged from approximately 41% to 65%, with higher reductions reported in selected smaller cohorts and case reports. Eldabe et al.^7^ conducted a multicenter RCT comparing PNFS plus optimized medical management (OMM) to OMM alone; one of the primary endpoints was lower back pain intensity reduction at 9 months. Lower back pain mean intensity reduction at 9 months was 47% on the VAS in the PNFS + OMM arm and 2.5% in the OMM alone arm. In a retrospective cohort of 20 patients implanted with PNFS, Schwarm et al.^31^ reported a median baseline pain score of 8.5/10 on NRS, which improved to 5/10 at 12 months postimplant, a 41.2% reduction in chronic back pain intensity. In a subsequent prospective study, Schwarm et al.^29^ evaluated PNFS in patients stratified by their response to prior transcutaneous electrical nerve stimulation (TENS). They found that TENS-responsive patients had a median NRS pain score improvement from 9/10 pre-PNFS to 4/10 at 6 months, whereas TENS nonresponders similarly improved from 8/10 to 4/10 after PNFS (both groups achieving substantial pain relief). Ishak et al.^12^ reported an average NRS rating improvement of 65% (from 8.5 to 3.0) at 24-month follow-up among 13 patients with PNFS implants. In a case series of patients with PSPS, D'Ammando et al.^4^ observed an average preimplant VAS pain score of 8.86, which decreased to 3.36 postimplant (approximately 62.1% reduction) in their cohort of 6 patients. Similarly, Patil et al.^26^ reported that in a 10-patient series, mean chronic back pain intensity dropped from 8.5/10 to 4.4/10 on the NRS after PNFS, corresponding to a 48.2% reduction in pain. In another prospective cohort, Schwarm et al.^30^ measured a median baseline NRS of 8.0, which improved to 4.5 (a 43.2% reduction, *P* < 0.001) at 3 months and further to 4.0 (50% reduction, *P* < 0.001) at 6 months post-PNFS.

**Table 3:** Pain reduction outcomes in chronic low back pain with peripheral nerve field stimulation.

Study	Intervention	Pain reduction outcome
Eldabe et al. (2019)^7^	PNFS + OMM vs OMM alone	Primary endpoint (9 mo, ITT): responder rate (≥50% pain relief): 33.9% in PNFS + OMM vs 1.7% OMM (*P* < 0.0001). Per-treatment: 56.7% vs 2.9%. Mean VAS (0–100 mm): 68.8 baseline → 36.9 at 9 mo (PNFS arm), vs 70.1 → 67.5 (OMM). % reduction: −47% vs −2.5% (*P* < 0.0001)
Rigoard et al. (2021)^28^	PNFS + SCS vs SCS only	Primary endpoint (3 mo): back pain surface ↓ 80.2% ± 21.3 with PNFS + SCS vs +13.2% ± 94.8 with SCS only (*P* = 0.012). Back pain VAS (3 mo): −68.8% ± 19.9 with PNFS + SCS vs +4.0% ± 15.0 with SCS only (*P* < 0.0001). At 1 mo, VAS −65.4% ± 20.4 vs +8.6% ± 26.6 (*P* = 0.001)
van Gorp et al. (2019)^39^,*	SCS + PNFS vs SCS (RCT 12 mo follow-up)	Primary endpoint (12 mo): back pain VAS (0–100): 73.0 → 43.5; Δ −30.0 mm (95% CI −37.7 to −22.4), *P* < 0.001. McGill PRI total: −18.3, *P* = 0.003
van Gorp et al. (2016)^40^,*	SCS + PNFS vs SCS as control	Primary endpoint (3 mo): ≥50% back pain relief: 42.9% PNFS vs 4.2% control (Δ 38.7%, *P* = 0.001). VAS back (0–100): PNFS: baseline 75.3 → 44.8 at 3 mo (Δ −27.2, *P* < 0.001); control: 68.9 → 71.4 (no change)
van Heteren et al. (2023)^41^,*	SCS + PNFS vs SCS.	SCS + PNFS (n = 54): legs 71.07 (11.90) → 29.06 (23.97), Δ −42.01 (3.20), *P* < 0.001; back 73.01 (10.02) → 44.23 (26.56), Δ −28.78 (3.68), *P* < 0.001. SCS-only (n = 21): legs 72.71 (13.63) → 21.64 (20.97), Δ −51.07 (5.20), *P* < 0.001; back 71.29 (12.22) → 42.97 (27.73), Δ −28.32 (6.05), *P* < 0.001. Between-group regression at 12 mo: no significant difference (back β = 1.09, *P* = 0.887)
van Roosendaal et al. (2023)^42^,*	SCS + PNFS vs SCS (subanalysis of van Heteren et al. RCT)	Back: 71.6 → 42.1 (SCS only, *P* < 0.001); 73.0 → 44.2 (SCS + PNFS, *P* < 0.001). No significant diff. between groups (*P* = 0.755). Leg: 72.9 → 19.8 (SCS only, *P* < 0.001); 71.1 → 29.1 (SCS + PNFS, *P* < 0.001). No sig. difference between groups (*P* = 0.117)
Ishak et al. (2018)^12^	PNFS	In responders, mean VAS: 8.5 baseline → 3.3 at 6 mo → 3 at 24 mo (*P* < 0.001). In nonresponders, mean VAS: 8 at baseline → 5.27 at 24 mo
Kloimstein et al. (2014)^14^	PNFS (alone or PNFS + SCS)	Mean VAS: 7.97 at baseline → 4.7 at 6 mo (≈44% reduction, *P* < 0.01). PNFS alone is slightly better than hybrid, not significant. ITT worst-case: 7.97 → 5.45; continuation: 7.97 → 4.61
Patil et al. (2014)^26^	PNFS	At median 24 mo: 6/10 patients (60%) achieved ≥50% pain relief; 2 patients (20%) achieved 20%–30% relief; 2 (20%) had no benefit. Examples: baseline NRS 7–10 → 0–5 in responders. Two patients became pain-free
Schwarm et al. (2021)^30^,†	PNFS	Median NRS: 8.0 baseline → 4.5 at 3 mo (*P* < 0.001) → 4.0 at 6 mo (*P* < 0.001). Improvement consistently regardless of baseline HADS/SCL-90 status
Schwarm et al. (2021)^29^,†	PNFS (TENS+ vs TENS− subgroups)	TENS+ cohort (n = 17): baseline NRS 9.0 → 5.0 after TENS (*P* < 0.001). PNFS trial: NRS 9.0 → 4.0 (*P* < 0.001). At 3 and 6 mo: NRS 4.0 (*P* < 0.001). ≥50% pain relief: 67% at trial, 67% at 3 mo, 60% at 6 mo. TENS− cohort (n = 24): baseline NRS 8.0 → no change after TENS. PNFS trial: NRS 8.0 → 4.0 (*P* = 0.004). At 3 mo: NRS 5.0 (*P* = 0.001), at 6 mo: NRS 4.0 (*P* < 0.001). ≥50% pain relief: 48% at trial, 33% at 3 mo, 40% at 6 mo
Schwarm et al. (2019)^31^,†	PNFS	Median NRS: 8.5 baseline → 5.3 at 6 mo (*P* = 0.0004) → 5.0 at 12 mo (*P* = 0.0002). Subgroup TENS+: baseline 8.5 → 3.0 at 6 mo → 5.0 at 12 mo. TENS−: baseline 8.25 → 5.5 at 6 mo → 5.0 at 12 mo
D'Ammando et al. (2016)^4^	PNFS	59% of patients (13/22) achieved sustained pain relief. Mean VAS decreased from 8.86 to 3.36 (Δ ≈ −5.5). Outcomes: excellent (75%–100% reduction) in 6, good (50%–74%) in 3, fair (25%–49%) in 4. Poor/no response in 9 patients
Hamm-Faber et al. (2015)^11^	SCS + PNFS, PNFS alone	Baseline: VAS leg 59 ± 15, low back 63 ± 14. At 12 mo: leg 20 ± 11, back 33 ± 16 (both *P* = 0.001). At 46 mo: leg 37 ± 17 (*P* = 0.027), back 40 ± 21 (*P* = 0.013). Significant long-term improvement
Chakrabortty et al. (2016)^3^	PNFS	Both patients achieved >50% pain relief during trial and maintained long-term benefit. Patient 1: comfortable for 2 y until death (unrelated cause). Patient 2: >5 y of stable analgesia with PNFS.
Granville et al. (2017)^9^	PNFS	VAS: 8–9 baseline → 0–1 during 10-d trial; pain returned to baseline (8–9) during 30-d stimulation-free period; postimplant: VAS 0–1 at 3 mo, sustained with daily use
Shah et al. (2022)^33^	PNFS	Baseline VAS 10 cm → 1 cm with subcutaneous leads (≈90% reduction). Pain relief sustained for 2 y at follow-up
Tokas et al. (2025)^37^	PNFS	Baseline: VAS 5/10 on SIJ palpation. Postimplant: significant pain relief immediately, completely pain-free a few days later, sustained at 6-mo follow-up

Summarizes pain relief achieved with PNFS or PNFS + SCS compared with control interventions. Pain outcomes are reported as mean percentage reduction in VAS or NRS scores or as responder rates (proportion achieving ≥50% pain reduction) from baseline.

Rows sharing the same superscript should not be interpreted as independent patient cohorts or summed across rows.

* Publications derived from the same multicenter randomized cohort; rows are retained because they report different timepoints, subgroup analyses, or stimulation-parameter data.

† Publications from overlapping single-center Schwarm cohorts; rows are retained because they report different analyses or outcome domains.

CLBP, chronic low back pain; IPG, implantable pulse generator; ITT, intention to treat; NRS, numeral rating scale; OMM, optimized medical management; PNFS, peripheral nerve field stimulation; PRI, patient review instrument; PSPS, persistent spinal pain syndrome; RCT, randomised controlled trial; SCS, spinal cord stimulation; SIJ, sacroiliac joint; TENS, transcutaneous electrical nerve stimulation; VAS, visual analogue scale.

**Figure 3.**
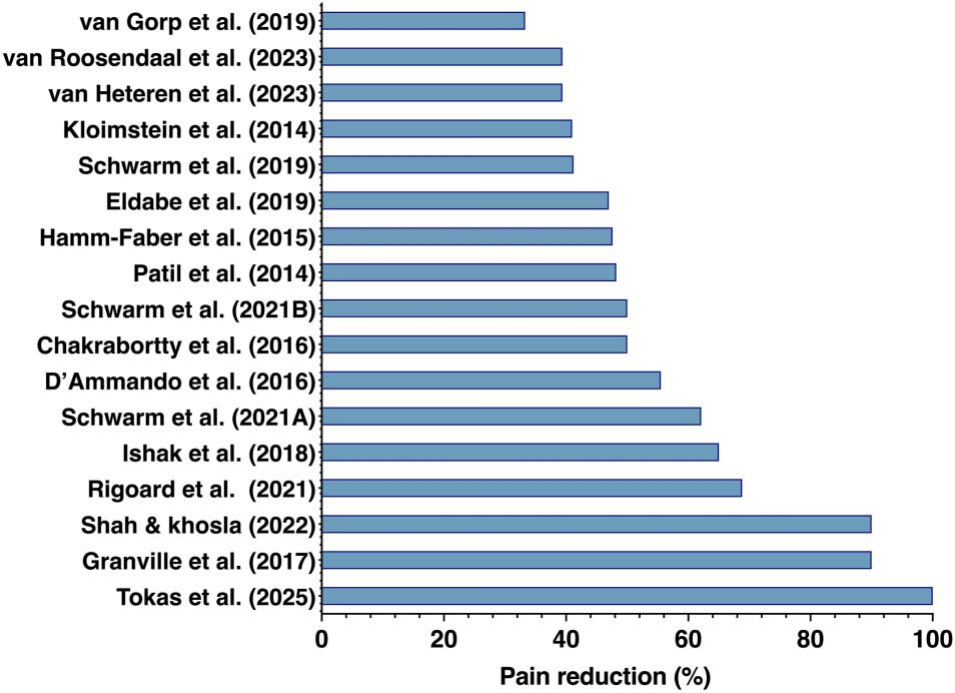
Pain reduction after PNFS. Graph summarizing the percentage reduction in pain scores (VAS or NRS) across all included studies of peripheral nerve field stimulation. Where applicable, outcomes for PNFS alone or PNFS + SCS are shown. Only studies reporting relevant data are presented. Single-patient and 2-patient case reports are shown as illustrative evidence only and were not used to derive summary ranges of treatment effect. Publications derived from overlapping cohorts should not be interpreted as independent estimates. NRS, numeral rating scale; PNFS, peripheral nerve field stimulation; SCS, spinal cord stimulation; VAS, visual analogue scale.

Four publications were single-patient or 2-patient case reports and were considered illustrative rather than primary evidence of efficacy.^3,9,33,37^ They described selected patients with focal lumbar, sacroiliac, or coccygeal pain, with reported outcomes including pain reduction from 8-9/10 to 0-1/10,^9^ approximately 90% pain relief in sacroiliac-region pain,^33^ near-complete pain relief at 6 months in coccydynia/SIJ pain,^37^ and >50% reduction in analgesic requirements in 2 SIJ pain cases.^3^ These reports provide useful technical and clinical context but were not used to derive summary ranges of treatment effect and should not be interpreted as generalizable efficacy estimates.

#### 3.7.2. Peripheral nerve field stimulation combined with spinal cord stimulation (hybrid therapy)

Seven included studies evaluated PNFS used in combination with SCS, typically to address axial low back pain that was not fully relieved by SCS alone. In studies reporting back-pain VAS change for PNFS combined with SCS, study-level reductions generally ranged from approximately 33% to 69%, with the largest effects observed in salvage or add-on contexts for patients with persistent axial pain despite SCS. Van Gorp et al.^40^ conducted an RCT comparing outcomes in patients implanted with SCS + PNFS vs those with SCS alone for 3 months and then added PNFS to the SCS group for the remaining 9 months. After 3 months, the SCS + PNFS group demonstrated a 41.3% decrease in median VAS (from a baseline of 75.3 to 44.8), whereas the SCS-only group showed a slight increase of 3.6% (from baseline 68.9 to 71.4). In a follow-up report, Van Gorp et al.^39^ observed that at 12 months, the SCS + PNFS group maintained a 33.3% VAS reduction (from 72.3 to 48.2). The SCS-only patients, who were subsequently given PNFS after the initial 3 months, experienced a larger mean reduction, from 73.9 to 38.2 (48.3%). These results are consistent with subgroup analyses by Van Heteren et al.^41^ and Van Roosendaal et al.^42^

Other studies have explored combined therapy in nonrandomized contexts. Hamm-Faber et al.^11^ followed 11 patients with SCS + PNFS implants for low back pain and reported a 47.6% reduction in VAS at 1 year, which slightly diminished to a 36.5% reduction at 4 years. In the large prospective multicenter study by Kloimstein et al.,^14^ at 6 months, the PNFS-only group had an average pain reduction of 44% VAS, whereas those who had combined SCS + PNFS therapy reported a similar pain reduction of about 41% from baseline. Interestingly, Rigoard et al.^28^ conducted a small crossover RCT (13 patients) evaluating PNFS as a salvage therapy for patients with persistent back pain despite SCS. After 3 months, the group receiving SCS + PNFS experienced a 68.8% decrease in VAS scores, whereas the group maintained on SCS alone had a slight increase (+4%) in back pain intensity over the same period. These findings indicate that adding PNFS to SCS does not consistently outperform SCS alone across all studies, but clinically meaningful benefits were observed in several studies, particularly in SCS nonresponders with persistent axial CLBP (Table 3).

### 3.8. Functional outcomes: Oswestry Disability Index

The impact of PNFS on functional disability because of low back pain was assessed in 9 of the included studies using the ODI, a validated questionnaire that quantifies disability on a scale from 0 (no disability) to 100 (maximal disability) (Table 4). In general, PNFS therapy was associated with meaningful improvements in ODI (ie, reductions in disability) (Fig. 4A). In their RCT, Eldabe et al.^7^ reported a mean ODI of 49.4 among all subjects at baseline, indicating a severe disability. At 9 months, PNFS + OMM patients had an absolute decrease of 9.8 points in ODI (improvement in disability), compared to essentially no change (−0.5 points) in the OMM-only group (intention-to-treat analysis). In a retrospective analysis of 25 PNFS cases for CLBP, Schwarm et al.^31^ reported a preoperative median ODI of 56, which improved to 42 at 6 months and 41 at 12 months postimplant, a significant functional improvement. Interestingly, Ishak et al.^12^ noted in their cohort that patients who responded positively to the trial stimulation phase had slightly higher baseline disability but went on to show a much larger ODI improvement 24 months after implantation than those who were trial-phase nonresponders (nonresponders to the trial who still underwent implantation did show some ODI improvement at follow-up, but it was far less pronounced than the 40.3 decrease seen among the responder group). In the RCT by Van Gorp et al.,^39^ patients receiving combined SCS + PNFS therapy had a mean ODI of 57.1 at baseline and improved to 40.4 at the 12-month follow-up (among 50 patients who completed 12 months), reflecting a significant reduction in disability with hybrid stimulation. A subgroup analysis of that trial by Van Heteren et al.^41^ found that both groups (SCS + PNFS and SCS alone) had less disability at 12 months compared with baseline: for the SCS + PNFS group (n = 54), the mean ODI decreased from 57.4 to 41.5, and the SCS-only group showed a similar magnitude of improvement with ODI reduction from 49.1 at baseline to 37.14 at 12 months. In their prospective multicenter study with 74 patients, Kloimstein et al.^14^ documented a significant mean ODI improvement (*P* < 0.01; *t* test) from 38.2 at baseline to 34.6 after 6 months of PNFS therapy. Schwarm et al.^30^ led a prospective study to evaluate psychological aspects of pain in 39 PNFS patients and measured disability: their preoperative median ODI was 59, improving to 45 at 3 and 6 months (*P* < 0.001), indicating a sustained reduction in disability within the first 6 months. In the Rigoard et al.^28^ salvage trial, the mean ODI in the SCS + PNFS add-on group improved from 50.3 at baseline to 37.3 at 3 months, whereas the control group (remaining on SCS alone) showed little change. Finally, in a study assessing the predictive value of TENS for PNFS outcomes, Schwarm et al.^30^ mentioned that in the TENS-responsive cohort (those who previously benefited from TENS), the median ODI improved from 60 at baseline to 46 at 3 and 6 months post-PNFS. The TENS-nonresponsive cohort had a baseline ODI of 58, improving to 44 at 3 and 6 months. Thus, both subgroups achieved a similar level of disability reduction by 6 months, suggesting that even patients who did not respond to transcutaneous stimulation can attain significant functional improvement with implanted subcutaneous stimulation.

**Table 4:** Disability, functional, and quality-of-life outcomes after peripheral nerve field stimulation therapy.

Study	Measures	Disability outcomes	Quality of life outcomes
Eldabe et al. (2019)^7^	ODI, EQ-5D-5L, SF-36, PGIC	Baseline ODI 51.9 ± 13.2; 9-mo ODI 42.1 ± 18.0; Δ −12.4 vs OMM −0.1 (*P* < 0.0001)	EQ-5D index: 0.40 baseline → 0.56 at 9 mo (+0.19, *P* = 0.0003), vs OMM no change. SF-36 mental score ↑ 3.8 points (*P* = 0.0062), physical score not significant. PGIC “much/very much improved”: 33.3% vs 1.7% (*P* < 0.0001)
Rigoard et al. (2021)^28^	ODI, EQ-5D-3L, HADS (anxiety/depression), MQS-III	Baseline ODI 50.3 ± 11.8; 3-mo ODI 37.3 ± 24.0; within-patient ODI −11.9% at 6 mo (*P* = 0.02) and −10.8% at 12 mo (*P* = 0.03); between-group at 3-mo trend favouring PNFS + SCS: −31.5% ± 34.1 vs +5.0% ± 29.7 (*P* = 0.07)	Between-group (3 mo): EQ-5D change +0.23 ± 0.33 vs +0.02 ± 0.17 (ns). Within-patient after PNFS (pooled groups): EQ-5D +0.19 at 6 mo (95% CI 0.04–0.33; *P* = 0.017); ns at 12 mo. HADS-anxiety: −2.1 at 6 mo (*P* = 0.03), −2.0 at 12 mo (*P* = 0.008); HADS-depression: no significant change
van Gorp et al. (2019)^39^,*	ODI, SF-36, PGIC, HADS-A/D, MQS-III	Baseline ODI 57.1 ± 11.0; 12-mo ODI 40.4 ± 18.7; Δ −16.8 (SE 4.3; 95% CI −25.8 to −7.6; *P* < 0.001)	SF-36 (baseline → 12 mo, Δ, *P*): physical functioning +17.7, *P* < 0.001; role-physical +19.6, *P* = 0.005; vitality +11.2, *P* < 0.001; pain +18.9, *P* < 0.001; social functioning +18.7, *P* < 0.001; general health +6.1, *P* = 0.035; mental health ns. PGIC: 3.3 (improved). HADS-anxiety: −1.1, *P* = 0.041; HADS-depression: −1.3, *P* = 0.144 (ns). MQS: −2.0, *P* = 0.017
van Gorp et al. (2016)^40^,*	ODI, EQ-5D, PGIC, SF-36, MQS	Baseline ODI 60.0 (range 36–82); 3-mo ODI 43.0 (8–70); Δ −7.9 (95% CI −16.4 to 0.6; *P* = 0.068)	EQ-5D: 0.2 → 0.8 in PNFS (Δ +0.2, *P* = 0.015) vs 0.6 in control. PGIC in PNFS groups was −0.6 points more improved than in control (*P* = 0.036). SF-36 “Health” subscore ↑ 25 → 75 in PNFS (*P* = 0.043), augmentations in other SF-36 domains. MQS drug scores: reduction greater in PNFS (not significant, *P* = 0.238)
van Heteren et al. (2023)^41^,*	ODI, SF-36, HADS, EQ-5D-3L, McGill Pain Questionnaire	Baseline ODI 57.37; 12-mo ODI 41.50 ± 18.98; Δ PNFS + SCS −5.87 (*P* < 0.001) vs SCS-only −11.95 (*P* = 0.004); between-group at 12 mo NS (β −0.237, *P* = 0.963)	SF-36: both groups improved similarly—eg, SCS + PNFS, significant gains in physical functioning (Δ +16.94, *P* < 0.001), social functioning (Δ +17.13, *P* < 0.001), bodily pain (Δ +17.73, *P* < 0.001), role-physical (Δ +18.98, *P* = 0.001), energy/fatigue (Δ +9.94, *P* = 0.001), general health (Δ +5.93, *P* = 0.030); SCS-only improved in physical functioning (*P* = 0.012), social functioning (*P* = 0.001), bodily pain (*P* = 0.039). HADS-anxiety: improved in SCS + PNFS (*P* = 0.048); depression ns. McGill: both improved on evaluative index; other indices improved only in SCS + PNFS. EQ-5D collected; no between-group difference at 12 mo on regression (β ≈ 0.013, *P* = 0.872)
van Roosendaal et al. (2023)^42^,*	ODI, no direct QOL measurements (those were in earlier RCT reports)	Same as van Heteren 2023: baseline ODI 57.37; 12-mo ODI 41.50 ± 18.98; no additional disability outcomes	Higher total charge/second in PNFS + SCS correlated with better QoL in a related analysis
Ishak et al. (2018)^12^	ODI, EQ-5D	Baseline ODI: responders ≈58, nonresponders ≈41; post-treatment ODI: responders ≈17.5 at 6 mo and 17.7 at 24 mo, nonresponders ≈32.4 at 24 mo; change Δ ≈ −41 points in responders and −8 points in nonresponders (*P* < 0.001)	In responders, EQ-5D index ↑ from 0.25 to 0.83 in 6 mo and 0.71 at 24 mo (*P* < 0.001). In nonresponders, EQ-5D index ↑ from 0.31 to 0.46 at 24 mo
Kloimstein et al. (2014)^14^	ODI, SF-12 (Physical and Mental); BDI	Baseline ODI 38.2 ± 7.05; 6-mo ODI 34.6 ± 7.77; Δ −3.6 (*P* < 0.01)	SF-12 improved (4.29 → 3.67, *P* < 0.01). BDI decreased (17.8 → 15.1, NS). Significant opioid/NSAID/anticonvulsant reduction also reported
Patil et al. (2014)^26^	Patient-reported (medication use, sleep pattern)	Baseline disability not systematically measured; post-treatment improvements inferred from reduced medication use and better sleep; qualitative improvement in most patients	6/10 patients reduced pain medication; 4/10 reported improved sleep. Not systematically quantified with standardized QoL tools
Schwarm et al. (2021)^30^,†	ODI, SF-12v2 PCS and MCS	Baseline ODI 59; post-treatment ODI 45 at 3 mo and at 6 mo (*P* ≤ 0.001); Δ −14 points	PCS: 29.3 baseline → 37.3 at 3 mo (*P* < 0.001) → 38.0 at 6 mo (*P* < 0.001). MCS: 36.2 baseline → 41.5 at 3 mo (*P* = 0.002) → 40.0 at 6 mo (*P* = 0.001). No differences between patients with high vs low baseline anxiety/depression
Schwarm et al. (2021)^29^,†	ODI, SF-12 PCS and MCS	Baseline ODI: TENS+ median 60 (IQR 48–68); TENS- median 58 (IQR 44–72); post-treatment ODI: TENS+ 46 at 3–6 mo, TENS− 44 at 3–6 mo; change −14 points (significant for TENS+)	PCS: TENS+: 26.4 → 37.2 (3 mo, *P* = 0.008) → 40.3 (6 mo, *P* = 0.001). TENS−: 31.2 → 37.9 (3 mo, *P* = 0.003) → 35.7 (6 mo, *P* = 0.017). MCS: TENS+: 41.1 → 49.5 (3 mo, *P* = 0.001) → 49.9 (6 mo, *P* = 0.004). TENS−: 31.2 → 33.2 (3 mo, ns) → 38.1 (6 mo, ns)
Schwarm et al. (2019)^31^,†	ODI, EQ-5D-5L, ASTS, CSQ-8	Baseline ODI median 56 (IQR 46–60); post-treatment ODI 42 at 6 mo and 41 at 12 mo; Δ ≈ −15 points (*P* = 0.001)	EQ-5D-5L: baseline 0.43 → 0.75 at 6 mo (*P* = 0.00005) → 0.76 at 12 mo (*P* = 0.0002). ASTS: ↑ positive mood (2.0 → 3.9 at 6 mo; 4.1 at 12 mo), significant ↓ sorrow, fatigue, anger. CSQ-8: median 29 at 12 mo (satisfied)
D'Ammando et al. (2016)^4^	Analgesic drug use as functional proxy	Baseline disability not quantified; responders (esp. PSPS, PHN, post-thoracotomy) showed functional improvement; qualitative improvement in ∼59% of patients	9 patients reduced drug use, 4 discontinued all analgesics, 9 had no change. QoL not systematically quantified but improved in responders
Hamm-Faber et al. (2015)^11^	QPDS, no generic QOL survey	Baseline QPDS 61 ± 15; post-treatment QPDS 49 ± 12 at 12 mo and 53 ± 15 at 46 mo; Δ −12 at 12 mo (*P* = 0.001) and −8 at 46 mo (*P* = 0.04)	No generic QoL survey, but disability improvement implies better functional QoL
Chakrabortty et al. (2016)^3^	Not specified (patient report)	Baseline disability not quantified; >50% functional improvement reported; qualitative significant improvement	Both patients reduced pain medications by half and reported >50% improvement in functional status
Granville et al. (2017)^9^	Patient-reported functional status	Baseline severe limitation: unable to sit and dependent on opioids/gabapentin; postimplant: fully active, medication-free and able to sit normally; >90% functional recovery	Patient resumed full activity, completely off medications, could sit comfortably; preferred bilateral cross-talk setting for greater coverage
Shah et al. (2022)^33^	NR	Baseline severe limitations requiring assistive devices; at 2-y follow-up: fully ambulatory and independent in ADLs; improved stair negotiation and walking without aids	NR
Tokas et al. (2025)^37^	Patient-reported outcomes	Baseline severe disability from 9 y of refractory pain; at 6 mo: fully independent and pain-free; complete resolution of disability	Marked improvement in daily life: postoperative independence, no social restrictions

This combined table lists baseline and post-treatment ODI scores and other functional scales, together with the absolute change (Δ) and significance where reported. It also summarizes changes in health-related quality-of-life metrics—including SF-36 or SF-12 domains, EQ-5D indices, and mental health scales such as the HADS-for each study. Values are presented as means ± SD or ranges.

Rows sharing the same superscript should not be interpreted as independent patient cohorts or summed across rows.

* Publications derived from the same multicenter randomized cohort; rows are retained because they report different timepoints, subgroup analyses, or stimulation-parameter data.

† Publications from overlapping single-center Schwarm cohorts; rows are retained because they report different analyses or outcome domains.

ADL, activities of daily life; BDI, Beck Depression Inventory; FBSS, failed back surgery syndrome; HADS, Hospital Anxiety and Depression Scale; IQR, interquartile range; MCS, mental component summary; NR, not reported; ODI, Oswestry disability index; OMM, optimized medical management; PCS, physical component summary; PHN, postherpetic neuralgia; PNFS, peripheral nerve field stimulation; QoL, quality of life; QPDS, Quebec Pain Disability Scale; RCT, randomised controlled trial; SCS, spinal cord stimulation; TENS, transcutaneous electrical nerve stimulation.

**Figure 4.**
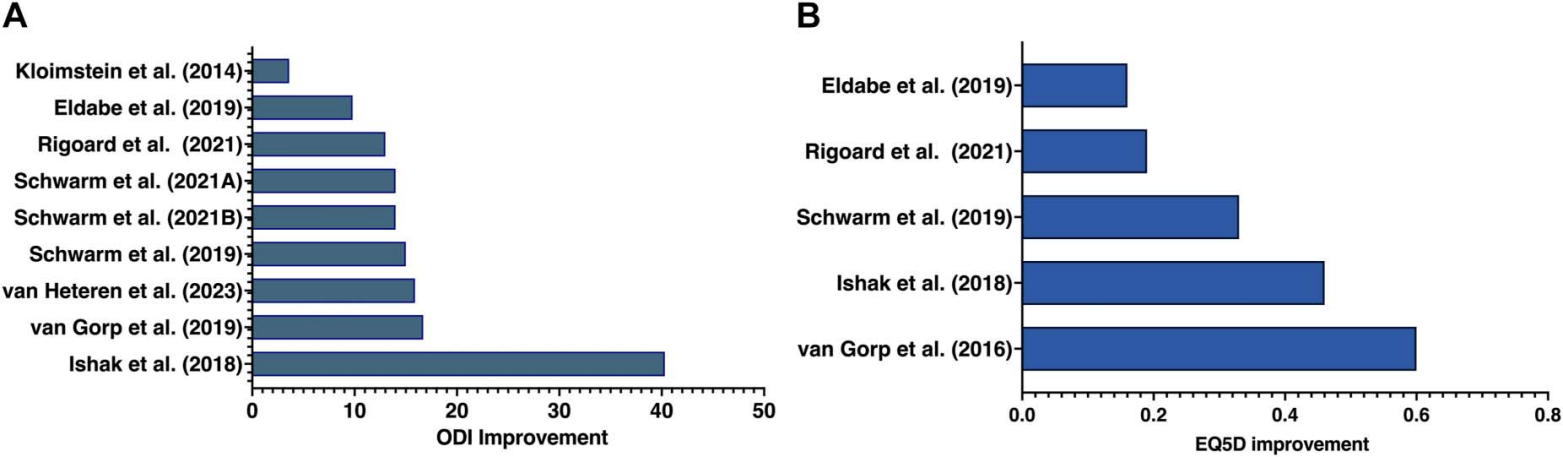
Disability and quality-of-life outcomes after PNFS. Panel A shows ODI improvement from baseline to follow-up across studies reporting ODI data. Panel B displays improvements in EQ-5D or health-related quality-of-life measures. Only studies reporting relevant data are presented. Publications derived from overlapping cohorts should not be interpreted as independent estimates. EQ-5D, EuroQol 5-Dimension; ODI, Oswestry Disability Index; PNFS, peripheral nerve field stimulation.

Overall, the ODI improvements observed (typically on the order of 10 to 20 points from baseline, up to 40.3 in 1 report^12^) are clinically meaningful, as a change of ∼10 points in ODI is often considered the minimal clinically important difference in chronic back pain populations (Table 4).

### 3.9. Quality of life outcomes: 36-Item Short Form Health Survey and others

Five of the included studies assessed QOL using the SF-36, which provides a Physical Component Summary and Mental Component Summary among other domain scores. The effects of PNFS on QOL were generally positive, although improvements in specific domains varied by study (Table 4). Eldabe et al.^7^ reported that compared with OMM-only group, PNFS + OMM group had a greater improvement in the mental component, but not in the physical component of the SF-36 at 9 months. Van Gorp et al.^39^ reported, at 12 months postimplant, notable improvements in several SF-36 subscales in the combined PNFS + SCS group: for example, the Physical Functioning score improved from a mean of 26.1 (baseline) to 43.3, and the Role-Physical (role limitations because of physical health problems) improved from 1.9 to 22.1. In a second analysis, Van Gorp et al.^38^ pooled data from 3 RCTs (including SubQStim and others) to identify predictors of therapy outcome. They found that in 1 RCT, a higher baseline physical health score was predictive of a better pain relief outcome with stimulation, whereas in 2 other trials, a higher baseline mental health score was a better predictor of success. This suggests that physical and psychological well-being might influence pain outcomes in some cases, although findings were not uniform across studies. Van Heteren et al.^41^ reported significant improvements in both physical and social functioning and pain components in both SCS + PNFS and SCS-only subgroups at 12 months. When directly comparing the 2 groups, the combined therapy cohort had greater improvement in certain domains, such as role limitations because of physical health, energy/fatigue, and general health, compared with SCS alone, whereas other domains improved similarly in both groups. In addition, Van Gorp et al.^40^ reported an augmentation of all SF-36 components within their PNFS-treated group.

Aside from SF-36, a few studies reported other QOL-related outcomes. Five studies used the EQ-5D (a standardized health utility index) (Table 4, Fig. 4B). Four of them reported an improved health-related QOL at follow-up.^7,12,31,40^ However, Rigoard et al.^28^ reported a significant increase between baseline and the 6-month follow-up but not between baseline and the 12-month follow-up. Patients in several studies also subjectively reported high satisfaction levels with their outcomes. Notably, Schwarm et al.^31^ reported a median CSQ-8 score of 29 at 12 months among their 20 implanted patients, which suggests patients' satisfaction with the treatment. These findings underscore that beyond pain relief, PNFS can confer meaningful improvements in mobility, daily function, and overall QOL for CLBP sufferers.

### 3.10. Complications

Peripheral nerve field stimulation is considered a relatively safe procedure, and no serious or life-threatening device-related complications (such as neural injury) were reported in the reviewed studies (Table 5). However, like all implantable neuromodulation therapies, PNFS is associated with certain hardware- and procedure-related complications. Across studies reporting patient-level complication rates, complications most commonly ranged from approximately 5% to 20%, although higher adverse-event burdens were reported in some longer-term or combined-therapy cohorts. The most common issues were lead migrations, local infections, and the need for revision surgeries (Fig. 5). In the RCT by Eldabe et al.,^7^ among 80 PNFS-implanted subjects, 5% had implant-related infections, 3.8% had a lead fracture, and 2.5% had a lead migration requiring repositioning. In their retrospective analysis of 25 patients implanted with PNFS devices for about 1 year, Schwarm et al.^31^ reported a 20% revision surgery rate. Three patients had a lead dislocation and 2 had infections because of stimulator implantation, this included 3 cases of lead dislodgement and 2 infections, all of which necessitated surgical intervention (either to reposition leads or remove the device in the case of infection). In 2 other studies by Schwarm et al.^29,30^ with 12-month follow-up, about 16% to 17% of patients with PNFS required revision: among 39 implanted patients, 3 had electrode migrations and 2 had infections that required hardware removal or replacement. Ishak et al.^12^ described a 23% complication rate among 13 implanted patients for 2 years: 1 patient developed a silicone allergy to the lead, 1 patient had a lead dysfunction, and 1 had a deep infection with wound discharge, requiring the device removal in those cases. In their RCT evaluating PNFS as an add-on to SCS for CLBP, van Gorp et al.^39^ reported 37 complications within 12 months among their 65 patients implanted with SCS + PNFS devices. The most frequent complications were pain at the implantable pulse generator site (n = 6), infections (n = 5), and PNFS lead dislocation (n = 4). In a large cohort of 105 PNFS implanted patients, Kloimstein et al.^14^ found a 9.6% complication rate over 6 months; this included 5 infections (4.8%) and 5 cases of loss of efficacy (4.8%), where the stimulation stopped providing benefit, which can prompt device removal or reprogramming. D'Ammando et al.^4^ stated that among 22 patients with various peripheral neuropathic pain syndromes treated with PNFS, 2 had infection and 2 others had painful muscular twitching or unpleasant sensation during stimulation. Patil et al.^26^ noted 2 complications among 10 implanted patients: 1 case of a flipped generator and 1 case of a migrated lead, both of which were corrected surgically. Hamm-Faber et al.^11^ documented 26 adverse events in 11 implanted patients within 4 years. Most of them were caused by hardware dysfunction or discomfort caused by implanted material. Some studies reported no implant-related complications, although safety reporting was inconsistent across smaller uncontrolled studies.

**Table 5:** Adverse events and complications of peripheral nerve field stimulation therapy.

Study	Complications reported
Eldabe et al. (2019)^7^	Across 80 implanted: 4 infections (5%), 3 lead fractures (3.8%), 2 lead migrations (2.5%). Other AEs: back/extremity pain, wound healing issues. No deaths, no lead erosion. Total of 193 AE, majority unrelated to device
Rigoard et al. (2021)^28^	33 AEs (4 severe). Most frequent: postop lead/IPG site pain 15.2%, falls 12.1%, early IPG battery depletion 9.1%, stimulation-induced fatigue 6.1%, digestive disorders 6.1%, others each 3.0% (nausea/headache; delayed wound healing; bursitis; lumbar hematoma; device displacement). No infections and no explants reported
van Gorp et al. (2019)^39^,*	At 12 mo: 37 AEs in 28/65 PNFS + SCS-treated patients; 12 patients (18.5%) required surgery. Infections: 5/65 (7.7%)—3 surgical (incl. 2 explants), 2 antibiotics only. Lead migration: epidural 4 (6.2%), PNFS 4 (6.2%); malfunctions reported. Pain at IPG site 6 (9.2%); connection/anchor site pain 4 total. Hematoma 2; acute lumbar disc herniation 1 (explant). Early battery depletion: 4 IPG replacements (≈6.2%). One death unrelated to therapy
van Gorp et al. (2016)^40^,*	In 65 randomized: 18 AEs (PNFS 8, control 10). Hardware: Octad lead migration 3 (4.6%), Quad lead migration 3 (4.6%), lead failures (n = 2). Biological: 2 infections (3.1%, 1 surgery, 1 antibiotics), 3 IPG/connection site pain (4.6%), 2 hematomas. 1 unrelated death. Overall, AE incidence not significantly different between groups
van Heteren et al. (2023)^41^,*	No separate safety data reported; this publication is a secondary analysis of the van Gorp randomized cohort
van Roosendaal et al. (2023)^42^,*	No separate safety data reported; this publication is a secondary analysis of the van Gorp randomized cohort
Ishak et al. (2018)^12^	The complication rate was 23% (3/13 patients). These were silicone intolerance, electrode dysfunction, and deep discharge. All 3 complications required surgical revision
Kloimstein et al. (2014)^14^	9.6% overall: infections (4.7%, n = 5), loss of efficacy because of lead migration/dislocation (4.7%, n = 5), skin irritation at IPG pocket (1 case). No major irreversible complications
Patil et al. (2014)^26^	1 flipped pulse generator, 1 lead migration, both were surgically revised. No infections, no neurological adverse events
Schwarm et al. (2021)^30^,†	Revision surgery 16.6%: 3 electrode dislocations, 2 infections. No neurological deficits
Schwarm et al. (2021)^29^,†	Revision surgery 16.6%: 3 electrode dislocations, 2 infections. No neurological deficits
Schwarm et al. (2019)^31^,†	Revision surgery in 20%: 3 lead dislocations, 2 infections. No neurological deficits reported
D'Ammando et al. (2016)^4^	1 lead infection, 1 connector cable infection, 1 painful muscle twitching during stimulation, 1 unpleasant stimulation sensation. No long-term severe complications
Hamm-Faber et al. (2015)^11^	Within 4 y: replacement of epidural lead (n = 2), PNFS connector problem (n = 1), connector site pain (n = 1), anchor-related pain (n = 1), IPG reposition (n = 3), recharging problems (n = 3), partial leg weakness after SIJ RF (n = 1). One explantation at 9 mo because of lack of efficacy
Chakrabortty et al. (2016)^3^	No procedure-related complications reported. One patient died 2 y later from unrelated medical causes
Granville et al. (2017)^9^	No procedure-related complications reported; no incisional pain; no AE over follow-up
Shah et al. (2022)^33^	No complications reported during implantation or follow-up
Tokas et al. (2025)^37^	No perioperative, early, or late complications observed

Details the types and frequencies of complications reported across studies, including lead migration, fracture or malfunction, infection, wound problems, reoperation rates, and other adverse events. The number and percentage of affected patients are listed, along with notable management and outcomes.

* Publications derived from the same multicenter randomized cohort. van Gorp et al. (2016) reports early randomized-phase adverse events, whereas van Gorp et al. (2019) reports 12-month follow-up safety data. van Heteren et al. (2023) and van Roosendaal et al. (2023) did not report separate complication data. Safety events from these overlapping publications were not summed across rows.

† Publications from overlapping single-center Schwarm cohorts. Safety data may overlap across these reports and were not summed as independent events.

AE, adverse event; IPG, implantable pulse generator; PNFS, peripheral nerve field stimulation; SIJ, sacroiliac joint.

**Figure 5.**
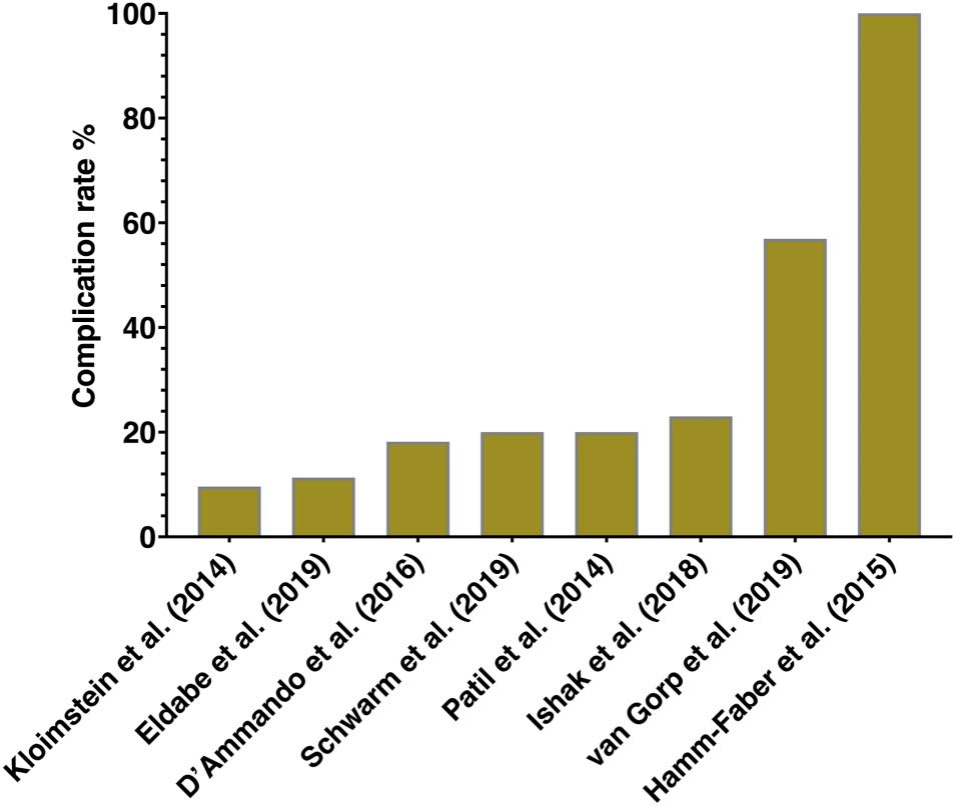
Reported complication rates after PNFS therapy. The chart depicts the proportion of patients with reported complications in studies providing patient-level complication rates. Complications included lead migration, lead fracture, infection, hardware malfunction, wound-related problems, and revision surgery. For overlapping cohorts, only 1 representative or most complete safety report was displayed to avoid double-counting. Secondary analyses without separate safety reporting were not shown separately. For studies reporting adverse-event counts without clear patient-level complication rates, values should be interpreted cautiously. PNFS, peripheral nerve field stimulation.

Although infections and lead-related issues do occur with PNFS, their reported rates appear broadly comparable to those described in the SCS literature; a meta-analysis reported an overall SCS complication rate of approximately 21%, with infection rates of 3.8% and lead migration of 5.6%.^2,18^ By comparison, occipital nerve stimulation has been associated with substantially higher complication and revision rates, including lead migration reported in up to 100% of cases and overall adverse events in up to 71% of patients.^15,20^ Although direct comparisons across anatomical targets should be interpreted cautiously, these data provide context for the comparatively favorable safety profile reported for PNFS in the lumbar region. These events were usually manageable (with antibiotics, lead revision, or device removal if necessary). The absence of neurological complications in these studies underscores the safety of the subcutaneous lead placement, which avoids the risks associated with epidural lead placement near the spinal cord. Nonetheless, patients should be counseled about the possibility of needing reoperations for lead adjustments or dealing with superficial infections as part of the long-term management of PNFS therapy.

### 3.11. Risk of bias

The risk of bias in the included studies is summarized in Figure 1, with domain-level RoB2 judgments and rationales for randomized trials provided in Supplementary Table 2 (available at https://links.lww.com/PR9/A440). The RCTs included were generally unblinded, which is explained by the nature of tonic stimulation, as patients could perceive whether stimulation was active or by using their handheld device. In addition, ethical considerations prevent a true placebo control beyond the trial stimulation phase. This lack of blinding could inflate subjective outcomes like pain ratings because of the placebo effect. In addition, many studies were industry sponsored or involved authors with neuromodulation affiliations, which can introduce bias in reporting. The observational studies often lacked control groups, making it difficult to distinguish treatment effects from placebo or natural history. Case reports and small case series were interpreted cautiously because they are vulnerable to publication bias and may overrepresent successful outcomes. We attempted to mitigate bias by including all available studies and assessing their quality (as described in Methods), but the overall evidence base consists of moderate-quality data. These limitations highlight the need for careful interpretation of the results and for more rigorous, blinded trials when it is possible in the future.

### 3.12. Certainty of evidence

The certainty of evidence for the critical outcomes is summarized in Table 6. Overall, certainty was rated as low for pain intensity and ODI outcomes and very low for EQ-5D outcomes. These ratings were mainly driven by risk of bias related to lack of blinding and subjective patient-reported outcomes, heterogeneity in study designs and follow-up durations, inconsistent outcome reporting, small sample sizes in several studies, and limited availability of controlled comparative data. Therefore, although the available evidence generally suggests improvement in pain and function after PNFS, confidence in the estimated magnitude of benefit remains limited.

**Table 6:** Grading of Recommendations Assessment, Development, and Evaluation summary of findings for critical outcomes.

Critical outcome	Studies contributing evidence	Summary of findings	Certainty of evidence	Main reasons for rating
Pain intensity, assessed by VAS or NRS	19 studies	Most studies reported reductions in pain intensity after PNFS, with many PNFS monotherapy studies describing approximately 40%–60% reductions from baseline. Comparative studies of PNFS combined with SCS showed mixed findings, with greater benefit in some salvage/add-on contexts but less consistent superiority over SCS alone	Low ⊕⊕◯◯	Downgraded for serious risk of bias, mainly because of limited blinding and subjective patient-reported outcomes; inconsistency and clinical heterogeneity across populations, interventions, comparators, and follow-up durations; and imprecision related to small samples in several studies
Disability, assessed by ODI	9 studies	ODI generally improved after PNFS or PNFS + SCS, with reported reductions often in the range of approximately 10–20 points from baseline, although the magnitude varied across studies and patient subgroups	Low ⊕⊕◯◯	Downgraded for serious risk of bias, heterogeneity in study design and follow-up, inconsistent reporting of baseline and follow-up ODI values, and imprecision because of limited sample sizes and incomplete controlled comparative data
Health-related quality of life, assessed by EQ-5D	5 studies	EQ-5D outcomes were reported less consistently than pain or ODI. Several studies described improvement after PNFS, but findings were not uniform across all time points, and some benefits were not sustained or not statistically significant at longer follow-up	Very low ⊕◯◯◯	Downgraded for serious risk of bias, inconsistency, indirectness because of variable populations and treatment contexts, and imprecision from small numbers of studies and limited reporting of complete EQ-5D data

Certainty of evidence was assessed using the GRADE framework for pain intensity, disability, and health-related quality of life in studies evaluating PNFS, alone or combined with SCS, for chronic low back pain. Certainty ratings considered risk of bias, inconsistency, indirectness, imprecision, and publication bias. Because of substantial heterogeneity, no pooled effect estimates were calculated.

EQ-5D, EuroQol 5-Dimension questionnaire; GRADE, Grading of Recommendations Assessment, Development, and Evaluation; NRS, numeric rating scale; ODI, Oswestry Disability Index; PNFS, peripheral nerve field stimulation; SCS, spinal cord stimulation; VAS, visual analogue scale.

## 4. Discussion

The evidence indicates that PNFS can substantially reduce CLBP intensity, whether used alone or in combination with SCS. At the study level, pain reductions of approximately 40% to 60% from baseline were commonly observed with PNFS monotherapy, whereas PNFS combined with SCS showed a broader range of improvement depending on whether it was used as upfront combined therapy or as salvage/add-on treatment. Although the direction of effect was generally favorable across study designs, the certainty of evidence remains limited, and the magnitude of benefit should be interpreted cautiously because of heterogeneity, risk of bias, and the subjective nature of pain outcomes. These findings suggest that PNFS may help target axial low back or sacroiliac-region pain that may be incompletely covered by SCS, rescuing patients who are partial or nonresponders to SCS. In practice, these findings support a tailored neuromodulation approach: one might start with SCS and, if significant axial back pain persists, add PNFS leads to broaden the pain coverage. These observations are consistent across multiple study designs, but the certainty of evidence remains low to very low (Table 6), and the findings should be interpreted as hypothesis-generating rather than definitive.

Another important observation is the opioid-sparing effect of PNFS. Multiple studies reported that patients were able to reduce their analgesic use after PNFS. In 1 report, 92% of patients reduced pain medication consumption and 46% of patients discontinued opioid analgesics altogether after PNFS therapy.^12^ Such reductions are extremely valuable given the risks of long-term opioid treatment, highlighting that beyond improving pain scores, PNFS can reduce reliance on pain medications and their side effects. Peripheral nerve field stimulation was also associated with meaningful functional improvements, with many patients transitioning from severe or moderate disability to mild or moderate levels as measured by the ODI. These changes exceeded the commonly cited minimal clinically important difference of approximately 10 points, suggesting real-world functional gains such as increased independence in daily activities.^8^

Although physical health domains of the SF-36 generally showed more consistent improvement, mental health benefits were also observed, possibly because regaining control over pain can alleviate associated anxiety and depression. Patients with better baseline psychological resilience may leverage pain relief to reengage in activities, whereas those with poor baseline mental health may require adjunctive psychological support alongside PNFS to achieve meaningful quality-of-life gains. High patient satisfaction rates (up to 87% satisfied overall) further corroborate the combined benefits of pain reduction, functional improvement, and tolerability.^12,43^

### 4.1. Technical considerations and optimization of peripheral nerve field stimulation

From a technical standpoint, PNFS is distinguished by the simplicity of its implantation and flexibility of programming. Unlike SCS, which requires placing leads in the epidural space near the spinal cord, PNFS leads are placed superficially in subcutaneous tissue over the region of pain. This minimally invasive approach can often be done with local anesthesia or mild sedation and carries a lower risk profile. The ease of lead placement allows clinicians to target discrete painful foci that do not necessarily follow dermatomal patterns, for example, focal pain over the SIJ or along a surgical scar can be directly covered by a subcutaneous electrode. Proper lead configuration and anchoring are important for optimal outcomes. Most studies found that 1 to 2 leads were sufficient to cover the typical low back pain region (with a third lead occasionally added for very broad or bilateral pain distributions). Leads have been arranged in parallel or in a crisscross array to maximize the stimulated field with minimal overlap. Although additional leads can broaden coverage, they also increase system complexity and battery demand. Securing the leads is crucial: although PNFS implantation is straightforward, careful anchoring helps prevent lead migration, one of the more common device-related complications reported with this therapy. However, the anchoring techniques were underreported in the literature.

Regarding stimulation parameters, almost all reported PNFS use conventional tonic stimulation at frequencies of tens of hertz, producing paresthesia in the painful area and potentially modulating nociceptive processing through segmental and peripheral mechanisms. Novel waveforms developed for SCS, such as high-frequency 10 kHz and burst stimulation, have not yet been systematically studied for PNFS. Whether these paradigms would confer additional benefit in the context of subcutaneous field stimulation remains an open question. The analgesic mechanism of PNFS is thought to rely primarily on segmental inhibition through Aβ fiber activation at mid-range frequencies,^19,27^ which differs from the proposed mechanisms of paresthesia-free SCS waveforms; moreover, high-frequency subcutaneous stimulation would impose substantially greater energy demands on implanted pulse generators. To date, conventional tonic stimulation remains the standard and clinically validated mode for PNFS.^19,27^

Another technical consideration is compatibility with SCS systems. Many patients receiving PNFS already have, or eventually receive, an SCS system (and vice versa). Ideally, a single implantable pulse generator can drive both SCS and PNFS leads, and some manufacturers offer dual-port devices to accommodate an epidural lead and a subcutaneous lead on 1 unit. Using 1 generator for combined SCS + PNFS therapy simplifies the implanted hardware (1 battery and controller) and may reduce overall cost, although careful coordination is required so that programming changes to 1 set of leads do not inadvertently affect the other.

### 4.2. Economic aspects

Chronic low back pain imposes not only a health burden on patients but also a substantial economic burden through direct health care costs and indirect costs such as lost productivity. Data specific to PNFS remain limited, but 1 analysis by Green et al.^10^ in patients with persistent spinal pain syndrome from the SubQStim study found that PNFS plus medical management, while initially more expensive than medical management alone, yielded improvements that translated into cost offsets over time. That analysis concluded PNFS can be cost-effective in this population over a horizon of several years.

When PNFS is used in addition to SCS, the economic equation becomes more complex because of the incremental costs of extra leads and longer operative times. If the combined approach yields significantly better outcomes for a given patient (as it did in some salvage cases of SCS nonresponders), the added cost may be justified. Cost-effectiveness for combined PNFS + SCS therapy, therefore, likely depends on careful patient selection, identifying those unlikely to achieve adequate relief with SCS alone who might benefit from upfront combined therapy.

A clear economic advantage of PNFS is its reversibility and adaptability of the trial phase. If a patient does not achieve sufficient relief from PNFS, the subcutaneous leads can be removed relatively easily (often under local anesthesia), limiting the wasted cost of an ineffective therapy. However, formal economic evaluations of PNFS are sparse, and further research is needed to gather real-world cost data and determine its long-term cost-effectiveness in managing CLBP.

### 4.3. Limitations

This systematic review has several limitations. First, the included studies were highly heterogeneous. The 19 studies varied widely in design, including RCTs, nonrandomized cohorts, case series, and case reports, as well as in patient populations, intervention context, follow-up duration, and outcome measures. This heterogeneity precluded a meaningful meta-analysis, including within apparently similar subsets, because comparable contrasts were limited by single-trial evidence, differences in study design, intervention context, follow-up timing, and outcome reporting. We, therefore, relied on narrative synthesis and structured tabulation, which introduces a risk of subjective interpretation. In addition, the review was not prospectively registered, and no formal protocol was published before completion, which should be considered when interpreting the reproducibility and transparency of the review process.

Second, the overall certainty of evidence was limited. Only 3 RCTs were available, and these had important limitations, including lack of blinding and relatively short follow-up. Many studies did not include control groups; therefore, improvements observed with PNFS must be interpreted cautiously in light of the natural history of chronic pain and potential placebo responses. Publication bias is also likely, as positive outcomes with PNFS may be reported more frequently than negative or null results, especially in small case series and case reports. Although we attempted to mitigate this by including all eligible consecutive case series, unsuccessful PNFS cases may remain underreported.

Furthermore, data reporting was inconsistent across studies. Not all reports provided detailed baseline characteristics, and follow-up intervals varied substantially, complicating direct comparisons of outcomes. Some subgroup analyses, including comparisons of combined therapy with SCS alone, were retrospective or not originally powered to evaluate PNFS specifically; these findings should, therefore, be considered hypothesis-generating rather than definitive. In several cases, data extraction was limited by the information available in the published text because raw outcome data, standard deviations, or confidence intervals were not consistently reported.

Finally, the literature search was limited to English and French publications, so relevant studies in other languages may have been missed. This review also focused on classical PNFS using conventional SCS-type hardware and did not evaluate newer dedicated PNS platforms or restorative neurostimulation systems, which have become increasingly available in some markets and represent important areas for future comparative research. Cost-effectiveness and patient satisfaction could not be formally quantified because of inconsistent reporting and were, therefore, addressed qualitatively. Despite rigorous systematic review methods, including independent study screening and data extraction, the narrative synthesis necessarily involves some interpretive judgment.

## 5. Conclusion

This systematic review suggests that PNFS, either alone or in combination with SCS, is a promising neuromodulation option for selected patients with CLBP, with reported improvements in pain intensity, functional status, analgesic consumption, and QOL. However, according to the Grading of Recommendations Assessment, Development, and Evaluation of the available studies, the certainty of the evidence remains low to very low, and the magnitude of benefit should be interpreted with caution given the heterogeneity, risk of bias, and predominance of observational data to date.

In cases where SCS alone fails to provide sufficient back pain control, adding PNFS appears particularly valuable. Although combined PNFS + SCS therapy did not consistently demonstrate superiority in primary outcomes in randomized trials, subgroup analyses and observational studies suggest that it can be an effective rescue strategy for selected patients. PNFS also appears to have a generally favorable safety profile, with no serious neurological complications reported and complication rates broadly comparable to those reported for SCS, although long-term safety data from large, controlled studies remain limited. The opioid-sparing effect reported in many studies further enhances its therapeutic appeal. However, limitations remain. The current literature is marked by study heterogeneity, small sample sizes, and a lack of standardized outcome reporting. In addition, cost-effectiveness analyses are scarce and require further investigation.

Given the growing interest in PNFS as an adaptable and minimally invasive neuromodulation technique, robust real-world studies with systematic long-term follow-up are needed, alongside larger randomized trials with rigorous methodology. Future studies should evaluate PNFS across diverse clinical contexts, including monotherapy, combination therapy, and staged interventions, while also addressing health economic outcomes and patient-reported measures. Personalized and adaptive programming strategies tailored to individual patient profiles also warrant dedicated investigation, particularly the impact of continuous vs dynamically adjusted stimulation parameters and the optimization of combined SCS and PNFS therapy. Although the current evidence should be interpreted cautiously because of study heterogeneity and methodological limitations, PNFS may offer meaningful clinical benefit for carefully selected patients when integrated within a structured framework that includes individualized treatment planning, systematic outcome monitoring, and ongoing reassessment of therapeutic value. Finally, head-to-head comparisons between classical PNFS and emerging neuromodulation technologies, including dedicated PNS platforms and restorative neurostimulation approaches, are needed to clarify the relative role of each modality in CLBP management.

## Disclosures

The authors have no conflicts of interest to declare.

## Supplemental digital content

Supplemental digital content associated with this article can be found online at https://links.lww.com/PR9/A440.

## Supplementary Material

## References

[1] Areias AC Costa F Janela D Molinos M Moulder RG Lains J Scheer JK Bento V Yanamadala V Cohen SP Correia FD. Impact on productivity impairment of a digital care program for chronic low back pain: a prospective longitudinal cohort study. Musculoskelet Sci Pract 2023;63:102709. 10.1016/j.msksp.2022.102709 36543719

[2] Blackburn AZ Chang HH DiSilvestro K Veeramani A McDonald C Zhang AS Daniels A. Spinal cord stimulation via percutaneous and open implantation: systematic review and meta-analysis examining complication rates. World Neurosurg 2021;154:132–43.e1. 10.1016/j.wneu.2021.07.077 34343680

[3] Chakrabortty S Kumar S Gupta D Rudraraju S. Intractable sacroiliac joint pain treated with peripheral nerve field stimulation. J Anaesthesiol Clin Pharmacol 2016;32:392–4. 10.4103/0970-9185.173336 PMC5009853 27625495

[4] D'Ammando A Messina G Franzini A Dones I. Peripheral nerve field stimulation for chronic neuropathic pain: a single institution experience. Acta Neurochir (Wien) 2016;158:767–72. 10.1007/s00701-016-2713-8 26858209

[5] Deer TR Esposito MF McRoberts WP Grider JS Sayed D Verrills P Lamer TJ Hunter CW Slavin KV Shah JM Hagedorn JM Simopoulos T Gonzalez DA Amirdelfan K Jain S Yang A Aiyer R Antony A Azeem N Levy RM Mekhail N. A systematic literature review of peripheral nerve stimulation therapies for the treatment of pain. Pain Med 2020;21:1590–603. 10.1093/pm/pnaa030 32803220

[6] Deer TR Mekhail N Provenzano D Pope J Krames E Leong M Levy RM Abejon D Buchser E Burton A Buvanendran A Candido K Caraway D Cousins M Dejongste M Diwan S Eldabe S Gatzinsky K Foreman RD Hayek S Kim P Kinfe T Kloth D Kumar K Rizvi S Lad SP Liem L Linderoth B Mackey S McDowell G McRoberts P Poree L Prager J Raso L Rauck R Russo M Simpson B Slavin K Staats P Stanton-Hicks M Verrills P Wellington J Williams K North R; Neuromodulation Appropriateness Consensus Committee. The appropriate use of neurostimulation of the spinal cord and peripheral nervous system for the treatment of chronic pain and ischemic diseases: the neuromodulation appropriateness consensus committee. Neuromodulation 2014;17:515–50. 10.1111/ner.12208 25112889

[7] Eldabe SS Taylor RS Goossens S Bouche B Gültuna I Green C Tinsley J Luyet PP Buchser E. A randomized controlled trial of subcutaneous nerve stimulation for back pain due to failed back surgery syndrome: the SubQStim study. Neuromodulation 2019;22:519–28. 10.1111/ner.12784 PMC6767391 29704437

[8] Goree JH Grant SA Dickerson DM Ilfeld BM Eshraghi Y Vaid S Valimahomed AK Shah JR Smith GL Finneran JJ Shah NN Guirguis MN Eckmann MS Antony AB Ohlendorf BJ Gupta M Gilbert JE Wongsarnpigoon A Boggs JW. Randomized placebo-controlled trial of 60-day percutaneous peripheral nerve stimulation treatment indicates relief of persistent postoperative pain, and improved function after knee replacement. Neuromodulation 2024;27:847–61. 10.1016/j.neurom.2024.03.001 38739062

[9] Granville M Brennan P Jacobson RE. Bilateral peripheral nerve field stimulation for intractable coccygeal pain: a case study using dual lead intercommunication. Cureus 2017;9:e1832. 10.7759/cureus.1832 PMC5760113 29333355

[10] Green C Eldabe SS Taylor RS Zahra M Eggington S. Resource use and cost of subcutaneous nerve stimulation versus optimized medical management in patients with failed back surgery syndrome: an analysis of the SubQStim study. Neuromodulation 2021;24:1033–41. 10.1111/ner.13405 33905144

[11] Hamm-Faber TE Aukes H Van Gorp EJ Gültuna I. Subcutaneous stimulation as an additional therapy to spinal cord stimulation for the treatment of low back pain and leg pain in failed back surgery syndrome: four-year follow-up. Neuromodulation 2015;18:618–22. 10.1111/ner.12309 25943093

[12] Ishak B Campos B Brunn H Unterberg AW Ahmadi R. Feasibility, safety, and efficacy of subcutaneous peripheral nerve field stimulation for the treatment of refractory low back pain: a two-year single-center study. Neuroscience 2018;387:38–47. 10.1016/j.neuroscience.2017.12.011 29248528

[13] Kaye AD Ridgell S Alpaugh ES Mouhaffel A Kaye AJ Cornett EM Chami AA Shah R Dixon BM Viswanath O Urits I Edinoff AN Urman RD. Peripheral nerve stimulation: a review of techniques and clinical efficacy. Pain Ther 2021;10:961–72. 10.1007/s40122-021-00298-1 PMC8586305 34331668

[14] Kloimstein H Likar R Kern M Neuhold J Cada M Loinig N Ilias W Freundl B Binder H Wolf A Dorn C Mozes-Balla EM Stein R Lappe I Sator-Katzenschlager S. Peripheral nerve field stimulation (PNFS) in chronic low back pain: a prospective multicenter study. Neuromodulation 2014;17:180–7. 10.1111/ner.12139 24320718

[15] Magis D Schoenen J. Advances and challenges in neurostimulation for headaches. Lancet Neurol 2012;11:708–19. 10.1016/S1474-4422(12)70139-4 22814542

[16] Manchikanti L Singh V Falco FJE Benyamin RM Hirsch JA. Epidemiology of low back pain in adults. Neuromodulation 2014;17:3–10. 10.1111/ner.12018 25395111

[17] McGuinness LA Higgins JPT. Risk-of-bias VISualization (robvis): an R package and Shiny web app for visualizing risk-of-bias assessments. Res Synth Methods 2021;12:55–61. 10.1002/jrsm.1411 32336025

[18] Mekhail NA Mathews M Nageeb F Guirguis M Mekhail MN Cheng J. Retrospective review of 707 cases of spinal cord stimulation: indications and complications. Pain Pract 2011;11:148–53. 10.1111/j.1533-2500.2010.00407.x 21371254

[19] Melzack R Wall PD. Pain mechanisms: a new theory. Science 1965;150:971–9. 10.1126/science.150.3699.971 5320816

[20] Montenegro MM Kissoon NR. Long term outcomes of occipital nerve stimulation. Front Pain Res 2023;4:1054764. 10.3389/fpain.2023.1054764 PMC10067723 37021077

[21] Moola S Munn Z Tufanaru C Aromataris E Sears K Sfetcu R Currie M Qureshi R Mattis P Lisy K Mu PF. Chapter 7: systematic reviews of etiology and risk. In: Aromataris E Munn Z, editors. JBI Manual for Evidence Synthesis. Adelaide, Australia: JBI; 2020. doi:10.46658/JBIMES-20-08.

[22] Munn Z Barker TH Moola S Tufanaru C Stern C McArthur A Stephenson M Aromataris E. A Methodological quality of case series studies: an introduction to the JBI critical appraisal tool. JBI Evid Synth 2020;18:2127–33. 10.11124/JBISRIR-D-19-00099 33038125

[23] Nahm FS. From the torpedo fish to the spinal cord stimulator. Korean J Pain 2020;33:97–8. 10.3344/kjp.2020.33.2.97 PMC7136297 32235009

[24] Oh TK Song IA. Prevalence and risk factors for persistent spinal pain syndrome type II following spinal surgery: a nationwide retrospective cohort study. Pain Physician 2024;27:555–63. 39621983

[25] Page MJ McKenzie JE Bossuyt PM Boutron I Hoffmann TC Mulrow CD Shamseer L Tetzlaff JM Akl EA Brennan SE Chou R Glanville J Grimshaw JM Hróbjartsson A Lalu MM Li T Loder EW Mayo-Wilson E McDonald S McGuinness LA Stewart LA Thomas J Tricco AC Welch VA Whiting P Moher D. The PRISMA 2020 statement: an updated guideline for reporting systematic reviews. BMJ 2021;372:n71. 10.1136/bmj.n71 PMC8005924 33782057

[26] Patil AA Otto D Raikar S. Peripheral nerve field stimulation for sacroiliac joint pain. Neuromodulation 2014;17:98–101. 10.1111/ner.12030 23441931

[27] Provenzano DA Heller JA Hanes MC. Current perspectives on neurostimulation for the management of chronic low back pain: a narrative review. J Pain Res 2021;14:463–79. 10.2147/JPR.S249580 PMC7899039 33628045

[28] Rigoard P Ounajim A Goudman L Bouche B Roulaud M Page P Lorgeoux B Baron S Nivole K Many M Adjali N Charrier E Rannou D Poupin L Wood C David R Héraud D Moens M Billot M. The added value of subcutaneous peripheral nerve field stimulation combined with SCS, as salvage therapy, for refractory low back pain component in persistent spinal pain syndrome implanted patients: a randomized controlled study (CUMPNS study) based on 3D-mapping composite pain assessment. J Clin Med 2021;10:5094. 10.3390/jcm10215094 PMC8584602 34768614

[29] Schwarm FP Ott M Nagl J Bender M Stein M Uhl E Maxeiner H Kolodziej MA. The predictive value of transcutaneous electrical nerve stimulation for patient selection in peripheral nerve field stimulation for chronic low back pain: a prospective study. Neuromodulation 2021;24:1051–8. 10.1111/ner.13244 32757257

[30] Schwarm FP Ott M Nagl J Leweke F Stein M Uhl E Maxeiner H Kolodziej MA. Preoperative elevated levels for depression, anxiety, and subjective mental stress have no influence on outcome measures of peripheral nerve field stimulation for chronic low back pain: a prospective study. Neuromodulation 2021;24:1042–50. 10.1111/ner.13368 33522693

[31] Schwarm FP Stein M Uhl E Maxeiner H Kolodziej MA. A retrospective analysis of 25 cases with peripheral nerve field stimulation for chronic low back pain and the predictive value of transcutaneous electrical nerve stimulation for patient selection. Neuromodulation 2019;22:607–14. 10.1111/ner.12890 30548104

[32] Sdrulla AD Guan Y Raja SN. Spinal cord stimulation: clinical efficacy and potential mechanisms. Pain Pract 2018;18:1048–67. 10.1111/papr.12692 PMC6391880 29526043

[33] Shah T Khosla A. Successful use of subcutaneous stimulation for bilateral sacroiliac joint pain. Cureus 2022;14:e31495. 10.7759/cureus.31495 PMC9750041 36532941

[34] Song JJ Popescu A Bell RL. Present and potential use of spinal cord stimulation to control chronic pain. Pain Physician 2014;17:235–46. 24850105

[35] Sterne JAC Savović J Page MJ Elbers RG Blencowe NS Boutron I Cates CJ Cheng HY Corbett MS Eldridge SM Emberson JR Hernán MA Hopewell S Hróbjartsson A Junqueira DR Jüni P Kirkham JJ Lasserson T Li T McAleenan A Reeves BC Shepperd S Shrier I Stewart LA Tilling K White IR Whiting PF Higgins JPT. RoB 2: a revised tool for assessing risk of bias in randomised trials. BMJ 2019;366:l4898. 10.1136/bmj.l4898 31462531

[36] Strand N D’souza RS Hagedorn JM Pritzlaff S Sayed D Azeem N Abd-Elsayed A Escobar A Huntoon MA Lam CM Deer TR. Evidence-based clinical guidelines from the American Society of Pain and Neuroscience for the use of implantable peripheral nerve stimulation in the treatment of chronic pain. J Pain Res 2022;15:2483–504. 10.2147/JPR.S362204 PMC9419727 36039168

[37] Tokas G Mavridis I Birbilis T. Treatment of chronic refractory coccydynia with peripheral nerve field stimulation: a novel case. Folia Med (Plovdiv) 2025;67:e127238. 10.3897/folmed.67.e127238 40270177

[38] van Gorp EJ Eldabe S Slavin KV Rigoard P Goossens S Mielke D Barolat G Declerck C Gilmore C Gültuna I Vissers KCP Tinsley J Likar R Luyet PP. Peripheral nerve field stimulation for chronic back pain: therapy outcome predictive factors. Pain Pract 2020;20:522–33. 10.1111/papr.12880 32145131

[39] van Gorp EJJAA Teernstra O Aukes HJ Hamm-Faber T Bürger K Kallewaard JW Spincemaille G Schapendonk JWLC Vonhögen L Bronkhorst E Vissers KCP. Long-term effect of peripheral nerve field stimulation as add-on therapy to spinal cord stimulation to treat low back pain in failed back surgery syndrome patients: a 12-month follow-up of a randomized controlled study. Neuromodulation 2019;22:970–7. 10.1111/ner.12776 29608807

[40] van Gorp EJJAA Teernstra OPM Gültuna I Hamm-Faber T Bürger K Schapendonk R Willem Kallewaard J Spincemaille G Vonhögen LH Hendriks JCM Vissers KCP. Subcutaneous stimulation as ADD-ON therapy to spinal cord stimulation is effective in treating low back pain in patients with failed back surgery syndrome: a multicenter randomized controlled trial. Neuromodulation 2016;19:171–8. 10.1111/ner.12385 26890014

[41] van Heteren EPZ van Roosendaal BKWP van Gorp EJJAA Bronkhorst EM Kallewaard JW Wegener JT Bürger K Teernstra OPM Buschman HPJ Hamm-Faber TE Vissers KCP. Spinal cord stimulation with additional peripheral nerve/field stimulation versus spinal cord stimulation alone on back pain and quality of life in patients with persistent spinal pain syndrome. Neuromodulation 2023;26:658–65. 10.1016/j.neurom.2021.11.010 35088732

[42] van Roosendaal BKWP van Heteren EPZ van Gorp EJ Bronkhorst EM Kallewaard JW Wegener JT Burger K Teernstra OPM Buschman HPJ Hamm-Faber T Vissers KCP. Subcutaneous stimulation as add-on therapy to spinal cord stimulation in patients with persistent spinal pain syndrome significantly increases the total electrical charge per second: aspects on stimulation parameters and energy requirements of the implanted neurostimulators. Neuromodulation 2023;26:666–75. 10.1016/j.neurom.2021.12.005 35279384

[43] Verrills P Vivian D Mitchell B Barnard A. Peripheral nerve field stimulation for chronic pain: 100 cases and review of the literature. Pain Med 2011;12:1395–405. 10.1111/j.1526-4637.2011.01201.x 21812906

[44] Wells GA Shea B O'Connell D Peterson J Welch V Losos M Tugwell P. The Newcastle-Ottawa Scale (NOS) for assessing the quality of nonrandomized studies in meta-analyses. Ottawa, ON, Canada: Ottawa Hospital Research Institute; 2000.

