# Supplementary material for: Peripheral nerve field stimulation for chronic low back pain: a systematic review
Source: Pain Rep. 2026 Oct 5;11(6):e1488. doi: 10.1097/PR9.0000000000001488 (PMC13641655; doi:10.1097/PR9.0000000000001488)
Supplement: Supplementary file 1 [file painreports-11-e1488-s001.pdf]

Supplementary Tables:

Supplementary Table 1. Database-specific search strategies.
Searches were conducted using free-text terms adapted to each database. No controlled-vocabulary terms such as MeSH or Emtree were applied.

Database	Date searched	Search strategy	Limits
MEDLINE/PubMed	April 5-8, 2025	("peripheral nerve field stimulation"[Title/Abstract] OR "subcutaneous stimulation"[Title/Abstract] OR "subcutaneous electrical stimulation"[Title/Abstract] OR "peripheral nerve stimulation"[Title/Abstract]) AND ("low back pain"[Title/Abstract] OR "chronic low back pain"[Title/Abstract] OR "lumbar pain"[Title/Abstract] OR "chronic lumbar pain"[Title/Abstract] OR "failed back surgery syndrome"[Title/Abstract] OR "FBSS"[Title/Abstract] OR "persistent spinal pain syndrome"[Title/Abstract] OR "PSPS"[Title/Abstract] OR "sacroiliac joint pain"[Title/Abstract])	English/ French; January 2014- April 2025
Embase	April 5-8, 2025	('peripheral nerve field stimulation':ti,ab OR 'subcutaneous stimulation':ti,ab OR 'subcutaneous electrical stimulation':ti,ab OR 'peripheral nerve stimulation':ti,ab) AND ('low back pain':ti,ab OR 'chronic low back pain':ti,ab OR 'lumbar pain':ti,ab OR 'chronic lumbar pain':ti,ab OR 'failed back surgery syndrome':ti,ab OR 'FBSS':ti,ab OR 'persistent spinal pain syndrome':ti,ab OR 'PSPS':ti,ab OR 'sacroiliac joint pain':ti,ab)	English/ French; January 2014- April 2025
Cochrane Library	April 5-8, 2025	("peripheral nerve field stimulation" OR "subcutaneous stimulation" OR "subcutaneous electrical stimulation" OR "peripheral nerve stimulation") AND ("low back pain" OR "chronic low back pain" OR "lumbar pain" OR "chronic lumbar pain" OR "failed back surgery syndrome" OR "FBSS" OR "persistent spinal pain syndrome" OR "PSPS" OR "sacroiliac joint pain")	English/ French; January 2014- April 2025
Université Laval Library	April 5-8, 2025	("peripheral nerve field stimulation" OR "subcutaneous stimulation" OR "subcutaneous electrical stimulation" OR "peripheral nerve stimulation") AND ("low back pain" OR "chronic low back pain" OR "lumbar pain" OR "chronic lumbar pain" OR "failed back surgery syndrome" OR "FBSS" OR "persistent spinal	English/ French; January 2014- April 2025

Database	Date searched	Search strategy	Limits
		pain syndrome" OR "PSPS" OR "sacroiliac joint pain")	

Abbreviations: FBSS = failed back surgery syndrome; PSPS = persistent spinal pain syndrome.

Supplementary Table 2. Domain-level RoB2 assessment for randomized trials. The Cochrane RoB2 tool was used to assess risk of bias in randomized controlled trials. Because some publications represented long-term follow-up or secondary analyses of the same randomized trial dataset, RoB2 judgments were organized at the trial level rather than repeated for each derivative publication. Judgments are reported across the five RoB2 domains, with brief rationales supporting the overall assessment.

Study / trial	D1: Randomization process	D2: Deviations from intended interventions	D3: Missing outcome data	D4: Measurement of outcome	D5: Selection of reported result	Overall RoB2 judgment	Rationale
Rigoard et al. 2021 / CUM PNS	Low risk	High risk	Low risk	Low risk	Low risk	Some concerns	The randomization process and reporting of outcomes were considered appropriate, and missing outcome data did not appear to substantially compromise interpretation. However, the active stimulation context and limited blinding created risk related to deviations from intended interventions, particularly because participants could perceive stimulation and expectations may have influenced treatment experience. Given the small randomized sample and crossover/salvage design, the overall judgment was

Study / trial	D1: Randomization process	D2: Deviations from intended interventions	D3: Missing outcome data	D4: Measurement of outcome	D5: Selection of reported result	Overall RoB2 judgment	Rationale
							considered to raise some concerns.
Eldabe et al. 2019 / SubQStim	Low risk	High risk	High risk	Some concerns	Low risk	High risk	The randomization process was adequately reported and the selected outcomes were clinically relevant. However, blinding was limited because active tonic stimulation can produce perceivable paresthesia, creating risk of deviations from intended interventions and expectancy effects. Missing outcome data and crossover/per-treatment analyses introduced additional concerns when interpreting treatment effects. Because pain, disability, and quality-of-life outcomes were patient-reported, outcome measurement was judged to raise some concerns.
Van Gorp et al. 2016 and related follow-up/sub-analyses	Low risk	High risk	High risk	Low risk	Some concerns	High risk	The randomized design was considered appropriate, but the intervention involved active neuromodulation with limited blinding and patient-perceived stimulation, creating risk of deviations from intended interventions. Missing outcome data and follow-up/subgroup analyses introduced concerns regarding the robustness of effect estimates over time. Outcome measurement was considered acceptable

Study / trial	D1: Randomization process	D2: Deviations from intended interventions	D3: Missing outcome data	D4: Measurement of outcome	D5: Selection of reported result	Overall RoB2 judgment	Rationale
							within the trial context, while selection of reported results raised some concerns because related publications included follow-up and secondary analyses of the same randomized dataset.

Abbreviations: RoB2 = Cochrane risk-of-bias tool for randomized trials; D1 = bias arising from the randomization process; D2 = bias due to deviations from intended interventions; D3 = bias due to missing outcome data; D4 = bias in measurement of the outcome; D5 = bias in selection of the reported result; SCS = spinal cord stimulation.
